# LC–MS-based absolute metabolite quantification: application to metabolic flux measurement in trypanosomes

**DOI:** 10.1007/s11306-015-0827-2

**Published:** 2015-07-09

**Authors:** Dong-Hyun Kim, Fiona Achcar, Rainer Breitling, Karl E. Burgess, Michael P. Barrett

**Affiliations:** 1 Wellcome Trust Centre for Molecular Parasitology, Institute of Infection, Immunity and Inflammation, College of Medical Veterinary and Life Sciences, University of Glasgow, Glasgow, G12 8TA UK; 2 Centre for Analytical Bioscience, School of Pharmacy, University of Nottingham, University Park, Nottingham, NG7 2RD UK; 3 Manchester Centre of Synthetic Biology for Fine and Speciality Chemicals, Manchester Institute of Biotechnology, Faculty of Life Sciences, University of Manchester, Manchester, M1 7DN UK; 4 Glasgow Polyomics, Wolfson Wohl Cancer Research Centre, College of Medical Veterinary & Life Sciences, University of Glasgow, Glasgow, G61 1QH UK

**Keywords:** Trypanosome, LC–MS, Absolute metabolite quantification, Metabolic flux, ^13^C-labelled *E. coli*

## Abstract

Human African trypanosomiasis is a neglected tropical disease caused by the protozoan parasite, *Trypanosoma brucei*. In the mammalian bloodstream, the trypanosome’s metabolism differs significantly from that of its host. For example, the parasite relies exclusively on glycolysis for energy source. Recently, computational and mathematical models of trypanosome metabolism have been generated to assist in understanding the parasite metabolism with the aim of facilitating drug development. Optimisation of these models requires quantitative information, including metabolite concentrations and/or metabolic fluxes that have been hitherto unavailable on a large scale. Here, we have implemented an LC–MS-based method that allows large scale quantification of metabolite levels by using U-^13^C-labelled *E.* *coli* extracts as internal standards. Known amounts of labelled *E. coli* extract were added into the parasite samples, as well as calibration standards, and used to obtain calibration curves enabling us to convert intensities into concentrations. This method allowed us to reliably quantify the changes of 43 intracellular metabolites and 32 extracellular metabolites in the medium over time. Based on the absolute quantification, we were able to compute consumption and production fluxes. These quantitative data can now be used to optimise computational models of parasite metabolism.

**Electronic supplementary material:**

The online version of this article (doi:10.1007/s11306-015-0827-2) contains supplementary material, which is available to authorized users.

## Introduction

Human African trypanosomiasis (HAT), also known as sleeping sickness, is a neglected tropical disease in sub-Saharan Africa caused by the protozoan parasite *Trypanosoma brucei* and transmitted by tsetse flies (Barrett et al. 2007; Brun et al. 2010). HAT is fatal if untreated, but currently used trypanocidal drugs are difficult to administer, can cause severe side effects and suffer from emerging parasite resistance (Delespaux and de Koning 2007; Vincent et al. 2012). Therefore, new drugs are urgently required to treat the disease, and improved knowledge of the metabolism of the parasite is necessary to facilitate drug development. Computational models can be used to guide our analysis of parasite physiology, and they have been used in understanding *T. brucei* metabolism, where it is hoped that they will play a critical role in developing optimised anti-parasite drugs (Bakker et al. 2010). However, such models require quantitative measurements, including data on concentrations and fluxes of metabolites, for their optimisation. Such data have so far been collected on a small scale, targeting individual reactions and metabolites (Achcar et al. 2013, 2012; Albert et al. 2005; Bakker et al. 2000, 1997; Kerkhoven et al. 2013), but a full understanding of parasite physiology would benefit from a more global quantitative assessment of metabolite dynamics.

LC–MS-based metabolomic studies enable simultaneous measurement of multiple small molecules in a biological system (Theodoridis et al. 2012; Vincent et al. 2012). The resulting data have been widely used to investigate the *relative* changes in intracellular metabolite concentrations for biomarker discovery (Denery et al. 2010; van der Kloet et al. 2012). However, a full understanding of cellular responses requires *absolute* metabolite concentrations, which can feed into quantitative computational models of metabolism.

LC–ESI/MS is a powerful analytical tool for quantification and identification of small molecules (metabolites) by providing retention time and exact molecular weights. However, it has some disadvantages, including nonlinear responses due to detector response and matrix effects (Annesley 2003). This drawback can lead to nonlinear calibration curves for quantification, so that true levels of intracellular metabolites are difficult to ascertain (Shi 2003). In addition, variations in instrumental response and degradation of metabolites of interest during sample preparation can also result in a biased quantitative result (Vuckovic 2012). In order to overcome these challenges, uniformly (U)-^13^C-labelled isotopomers of metabolites can be added as internal standards, behaving identically to their unlabelled equivalents in sample extracts. The demonstrated fidelity of Orbitrap mass spectrometers for quantifying isotope ratios (Xu et al. 2010) makes them particularly suitable for this purpose. However, since the availability of commercial U-^13^C-labelled isotopomers is limited and they are often prohibitively expensive, in vivo synthesis of U-^13^C-labelled compounds is required using suitable microorganisms grown in U-^13^C-labelled glucose-limited culture media. Absolute intracellular concentrations of metabolites in a sample can then be calculated by adding known amounts of U-^13^C-labelled cell extract prior to the extraction procedure (Bennett et al. 2009; Mashego et al. 2004). The most suitable method for this isotope ratio-based MS (IR-MS) technique is to use the same organism for generating U-^13^C-labelled cell extracts which can cover all of intracellular metabolites for absolute quantification (Mashego et al. 2004; Wu et al. 2005). However, *T. brucei* cannot be used for this purpose, as media containing only glucose as carbon source are not available (Creek et al. 2013); hence, it is not possible to obtain all metabolites present in the cell in fully labelled form. Furthermore, *T. brucei* reaches a relatively low cell density when cultivated. Consequently, we decided to explore the utility of U-^13^C-labelled *E. coli* extract as an alternative source of isotope analogues for quantitative analysis of trypanosome metabolites.

In this paper, we report the first application of the IR-MS technique using U-^13^C-labelled *E. coli* extracts for the global quantitative analysis of intra- and extra-cellular metabolite concentrations in trypanosomes. We analyse the overlap of *E. coli* and *T. brucei* metabolomes to demonstrate the viability of the approach. Furthermore, by measuring absolute concentrations of extracellular metabolites in spent media, we were able to compute metabolic fluxes.

## Materials and methods

### Preparation of U-^13^C-labelled *E. coli* extract and calibration standards

*E. coli* was cultured on 100 % U-^13^C-labelled glucose and the labelled metabolites were extracted using existing sampling procedures (Rabinowitz and Kimball 2007; Winder et al. 2008). Briefly, *E. coli* MG1655 was grown in 500 ml of M9 minimal medium which contained the following per litre: 100 ml of 5 × M9 minimal medium (33.9 g of Na_2_HOP_4_, 15 g of KH_2_PO_4_, 2.5 g of NaCl), 1 ml of 1 M MgSO_4_, 1 ml of 1 M CaCl_2_, 3 ml of 2 M (NH_4_)_2_SO_4_ and 5 ml of 20 % U-^13^C-labelled glucose at 37 °C overnight. The culture was grown up to an optical density at 600 nm (OD_600_) of ~1.0 and the cells were centrifuged at 3000×*g* for 10 min. The supernatant was removed and 8 ml of −48 °C acetonitrile/methanol/water (2:2:1) was added into the cell pellet for quenching metabolism and extracting metabolites. Then, cells were freeze–thaw extracted (i.e., flash frozen in liquid N_2_ for 1 min and thawed at 4 °C, then vortexed for 30 s × 4) and centrifuged at 3000×*g* for 10 min. The supernatant was collected and stored at −80 °C prior to analysis. The U-^13^C-labelled extract provides a range of U-^13^C-labelled internal standards for each intracellular metabolite being investigated. Furthermore, absolute metabolite concentrations were obtained by adding the same amounts of the labelled *E. coli* extract to both cell samples prior to the extraction procedure and the unlabelled calibration standards.

The calibration standards for the absolute quantification of metabolites were prepared by diluting main stock solutions of unlabelled compounds obtaining seven concentration points (0.001, 0.01, 0.1, 1, 10, 50 and 100 µM or 0.03, 0.3, 3, 30, 300, 600, 1200 and 3000 µM). The standard samples were split into five groups according to their masses (see Supplementary Table S2), so that metabolites of similar mass do not overlap, and a clear and single peak of each metabolite in the MS data can be provided for calibration curves. Then, the U-^13^C-labelled *E. coli* extract was added to each calibration sample in a 1:4 ratio.

### Parasite growth conditions and metabolite extraction

For intracellular concentrations, *T. brucei* wild type strain 427 bloodstream forms were cultured in HMI-9 (Hirumi et al. 1977) or Creek’s minimal medium (CMM) (Creek et al. 2013) supplemented with 10 % fetal bovine serum at 37 °C, 5 % CO_2_ 2 × 10^4^ cells/ml were seeded in T150 culture flasks and allow them to reach a density of 2 × 10^6^ cells/ml. Appropriate volumes of cell culture were taken to yield 5 × 10^7^ cells and quenched in EtOH/dry ice bath as previously described (t’Kindt et al. 2010). Cells were centrifuged at 1250×*g* for 10 min and then the growth medium was removed and the cells were washed with 1 ml of PBS pre-cooled at 4 °C. The cell solution was centrifuged again at 1250×*g* for 5 min and the supernatant was removed. Metabolites were extracted by adding 100 µl of chloroform/methanol/water (1:3:1) spiked with the U-^13^C-labelled *E. coli* extract in a 1:4 ratio and mixed vigorously for 1 h at 4 °C. After centrifugation at 13,000×*g* for 10 min, the supernatant was collected and stored at −80 °C until LC–MS analysis. One pooled quality control (QC) sample was prepared by mixing all the samples in order to assess instrument performance. All samples were prepared in four biological replicates.

For extracellular concentrations, 0.38 × 10^4^ cells/ml were seeded in 40 ml of CMM or HMI-9 and incubated at 37 °C, 5 % CO_2_ for 57 h. The cells were sampled for analysis after 0, 7, 24, 31, 48, 51 and 57 h. During the incubation period, 5 ml of cell culture was taken and cells were counted. Then, the cell culture was centrifuged at 1250×*g* for 10 min and 10 µl of supernatant (spent medium) was taken for extracellular concentration measurements. The harvested spent medium was extracted by adding 190 µl of chloroform/methanol/water (1:3:1) spiked with the U-^13^C-labelled *E. coli* extract in a 1:4 ratio and mixed vigorously, then stored at −80 °C prior to analysis. All samples were prepared in five biological replicates.

### Analytical methodologies

Separations were performed on a Dionex UltiMate 3000 RSLC liquid chromatography system (Dionex, Camberley, Surrey). Chromatography was carried out using a ZIC-HILIC or ZIC-pHLIC 150 × 4.6 mm^2^, 5 µm column (Merck Sequant) as previously described (Creek et al. 2011). Briefly, the column was maintained at 30 °C and samples were eluted with a linear gradient from 80 % B to 20 % B over 32 min, followed by an 8 min wash with 5 % B, and 8 min re-equilibration with 80 % B at the flow rate of 300 µl/min. Mobile phase A was water (0.1 % v/v formic acid) for a ZIC-HILIC or 20 mM ammonium carbonate in water for a ZIC-pHILIC column and mobile phase B was acetonitrile (0.08 % v/v formic acid) for a ZIC-HILIC or 100 % acetonitrile for a ZIC-pHILIC column. The injection volume was 10 µl and samples were maintained at 4 °C. For the MS analysis, an Orbitrap Exactive (Thermo Fisher Scientific, Hemel Hempstead, UK) with a HESI-II probe was operated in polarity switching mode, with the following settings as previously described (Creek et al. 2011): resolution 50,000, AGC 1 × 10^6^, m/z range 70–1400, sheath gas 40, auxiliary gas 20, sweep gas 1, probe temperature 150 °C, and capillary temperature 275 °C. For positive mode ionisation: source voltage +4 kV, capillary voltage +50 V, tube voltage +70 kV, skimmer voltage +20 V. For negative mode ionisation: source voltage −3.5 kV, capillary voltage −50 V, tube voltage −70 V, skimmer voltage −20 V. Mass calibration was performed for each polarity immediately before each analysis batch. The calibration mass range was extended to cover small metabolites by inclusion of low-mass contaminants with the standard Thermo calibration mixture masses (below m/z 1400), C_2_H_6_NO_2_ for positive ion electrospray ionisation (PIESI) mode (m/z 76.0393) and C_3_H_5_O_3_ for negative ion electrospray ionisation (NIESI) mode (m/z 89.0244). To enhance calibration stability, lock-mass correction was also applied to each analytical run using these ubiquitous low-mass contaminants.

### Data processing and analysis

Raw LC–MS data were processed with XCMS for untargeted peak-picking (Tautenhahn et al. 2008) and mzMatch for peak matching and annotation of related peaks (Scheltema et al. 2011). Noise filtering and putative metabolite identification was performed by IDEOM using the default parameters (Creek et al. 2012). Metabolite identification was performed by matching accurate masses and retention times of authentic standards [Level 1 metabolite identification according to the metabolomics standards initiative (Sumner et al. 2014, 2007)], but when standards were not available, predicted retention times were employed, hence these identifications should be considered as putative (Level 2 identification).

For the extracellular medium quantifications, raw LC–MS data were processed with mzMatch and mzMatch-ISO (Chokkathukalam et al. 2013) (see R script in Supplementary File A). The peaks matching accurate mass and retention time with our calibration standards were then selected for interpretation.

### Estimation of metabolic fluxes

Among the 192 calibration standards examined, 32 had sufficiently clear single peaks in the *T. brucei* extracts, in the calibration standard and in the labelled *E. coli* extracts to allow absolute quantification. Calibration curves for these were constructed by calculating the ratio between the unlabelled U-^12^C peaks (from the standards) and the U-^13^C-labelled peaks (from *E. coli*). Using these calibration curves and the U-^12^C over U-^13^C ratios calculated from the spent medium time course (cells grown in CMM media), absolute concentrations of these 32 metabolites were calculated (see Sect. 3). Absolute concentrations of eight metabolites were also quantified in the spent medium of cells grown in HMI-9 medium for comparison. From these time courses, production or consumption fluxes could be estimated, assuming that these fluxes are constant over time. Supposing exponential growth, Eq. 1 expresses the cell density as a function of time:

1 $$c\left( t \right)\; = \;c_{0} \times \;\exp \left( {g\; \times \;t} \right),$$

where *c*_0_ is the cell density at time t = 0, *g* is the growth rate.

Assuming constant consumption/production flux, Eq. 2 describes the concentration of a metabolite *m* as a function of time *t*:

2 $$m\left( t \right)\; = \;\left( {k \times \frac{{c_{0} }}{g}} \right) \times \;\left( {\exp \left( {g \times \;t} \right)\; - \;1} \right)\; + m_{0} ,$$

where *k* is the flux and *m*_*o*_ is the concentration of metabolite *m* at time t = 0. Given Eqs. 1 and 2, the concentration of a metabolite *m* as a function of cell density can be expressed by Eq. 3:

3 $$\begin{array}{*{20}l} {m\left( t \right)\; = \;a \times \;c\left( t \right)\; + \;b} \\ {a\; = \frac{k}{g},\;b\; = - k \times \frac{{c_{0} }}{g}\; + m_{0} } \\ \end{array}$$

Parameters *g* and *c*_0_ were estimated by fitting the equation to the data using the python package lmfit (http://cars9.uchicago.edu/software/python/lmfit/) with the parameters g and *c*_0_ bounded below at 0. Similarly, *k* and *m*_0_ were estimated via *a* and *b* by fitting Eq. 3. The flux *k* was then converted from nmol/(h cell) to nmol/(min 10^8^ cells).

## Results and discussion

### Comparison of the *E. coli* and *T. brucei* metabolomes

**Fig. 1:**
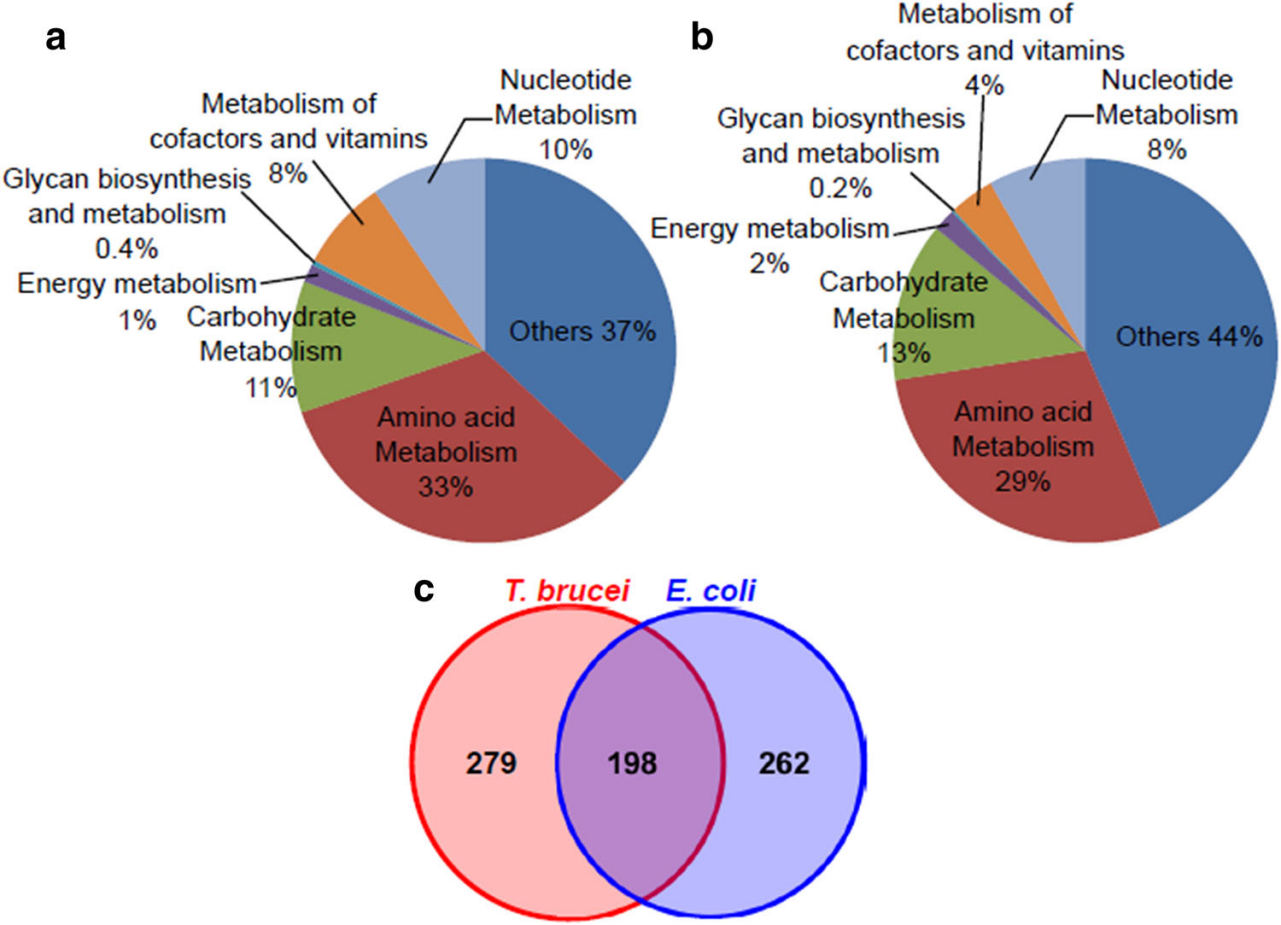
Comparison of the measured metabolomes extracted from *E. coli* (**a**) and *T. brucei* (**b**), and total number of metabolites identified (**c**). In total, 460 and 477 metabolites were putatively identified in *E coli* and *T. brucei* extracts, respectively, using LC–MS-based metabolite profiling. 198 metabolites were determined as common chemical structures between both species

The metabolomes of *E. coli* and *T. brucei* were investigated by an untargeted LC–ESI/MS technique to evaluate which metabolites can be quantified by the IR-MS technique. The observed chemical compositions of both species are shown in Fig. 1a, b.

Totals of 460 and 477 metabolites were putatively identified in *E. coli* and *T. brucei* extracts, respectively. Detected intracellular metabolites were dominated by a few chemical classes in both species: metabolites predicted to be related to the metabolism of amino acids (*E. coli*, 32.2 %; *T. brucei*, 28.7 %), carbohydrates (*E. coli*, 10.2 %; *T. brucei*, 12.6 %), nucleotides (*E. coli*, 8.0 %; *T. brucei*, 7.5 %), and cofactors and vitamins (*E. coli*, 8.2 %; *T. brucei*, 3.6 %). With the exception of compounds which were not assigned to any specific class of metabolites, amino acid metabolism-related metabolites were the most numerous compounds detected in the both extracts, followed by compounds involved in carbohydrate metabolism. Of the measured metabolites in the both extracts, 198 metabolites identified by the technique were common, as shown in Fig. 1c, and thus are potentially amenable to absolute quantification as described here. Many core metabolites, such as amino acids, carbohydrates and nucleotides, were cross-detected in both species, confirming our ability to investigate central metabolism of trypanosomes by measuring metabolic fluxes using the IR-MS technique with easily generated labelled *E. coli* extracts. The complete list of the common putative metabolites is available in Supplementary Table S3.

### Absolute quantitative analysis of intracellular metabolites in *T. brucei* using LC–ESI/MS

An absolute quantitative analysis of intra- and extra-cellular metabolites in *T. brucei* was carried out. To determine intracellular concentrations of metabolites in the parasite, sets of calibration standard mixtures of known concentrations added with U-^13^C-labelled *E. coli* extract in a 1:4 ratio were analysed by LC–ESI/MS. Calibration curves of each metabolite containing a minimum of five concentration points were then obtained by comparison of the ratios of mass spectral area from unlabelled (M + 0) and labelled {M + (N × 1.0033)} metabolites, where M is the monoisotopic mass (^12^C−) and N is the number of carbon atoms of the molecule. Coefficients of determination showed very good linearity of the calibration curves of 43 intracellular metabolites (R^2^ > 0.99, data not shown).

The absolute concentrations of 43 intracellular metabolites were determined against the calibration curves. The concentrations of intracellular metabolites are shown in Table 1. Of 43 measured intracellular metabolites, l-alanine was the most abundant compound (21.4 mM—HMI-9; 32.8 mM—CMM) in the cell extract, followed by pyruvate (7.2 mM—HMI-9; 7.1 mM—CMM), l-proline (3.8 mM—HMI-9; 2.7 mM—CMM) and spermidine (3.5 mM—HMI-9; 4.2 mM—CMM). The total concentration of the less abundant metabolites (50 % of all metabolites measured) was only 0.6 mM.

**Table 1:** The concentrations of intracellular metabolites in *T. brucei* grown in HMI-9 and CMM

Name	Concetration in HMI-9 (µM ± SEM)	Concetration in CMM (µM ± SEM)
*N*-Acetylornithine	0.2 ± 0.1	0.2 ± 0.0
cis-Aconitate	0.3 ± 0.0	0.3 ± 0.0
Adenosine	28.2 ± 2.2	12.2 ± 1.3
S-Adenosylmethionine	38.5 ± 2.9	44.7 ± 1.2
l-Alanine	21439.1 ± 246.8	32754.3 ± 1334.0
4-Aminobutanoate	19.4 ± 1.8	21.5 ± 2.4
l-Arginine	777.8 ± 28.9	1065.8 ± 111.9
l-Asparagine	2886.0 ± 96.2	2595.4 ± 121.8
L-Aspartate	395.0 ± 37.6	609.2 ± 80.2
Citrate	53.8 ± 5.7	57.7 ± 4.0
Fumarate	347.2 ± 44.4	305.0 ± 40.5
l-Glutamate	2769.4 ± 130.8	3502.7 ± 396.3
l-Glutamine	2469.5 ± 220.5	1736.3 ± 111.8
Glycine	3498.8 ± 94.5	2987.6 ± 58.9
l-Histidine	245.5 ± 8.1	238.4 ± 5.5
(R)-2-Hydroxyglutarate	128.6 ± 4.0	109.8 ± 4.7
Hypoxanthine	325.5 ± 31.5	1.4 ± 0.1
l-Isoleucine	299.2 ± 25.1	82.1 ± 10.4
l-Lysine	346.2 ± 40.5	133.2 ± 5.3
(S)-Malate	324.8 ± 36.2	287.2 ± 40.6
l-Methionine	156.9 ± 6.5	112.1 ± 5.6
5′-Methylthioadenosine	2.1 ± 0.2	2.3 ± 0.1
Nicotinamide	25.6 ± 2.9	14.5 ± 0.3
l-Ornithine	407.3 ± 25.4	1072.8 ± 58.5
Orotate	<0.3	<0.3
2-Oxoglutarate	845.9 ± 35.1	944.3 ± 131.3
Pantothenate	6.7 ± 0.5	0.3 ± 0.1
l-Phenylalanine	131.3 ± 12.0	48.2 ± 3.1
Phenylpyruvate	92.5 ± 3.8	51.1 ± 2.7
3-Phospho-D-glycerate	2251.7 ± 60.7	1101.1 ± 110.8
Phosphoenolpyruvate	52.6 ± 4.0	27.0 ± 2.2
l-Proline	3835.8 ± 277.2	2688.7 ± 177.5
Putrescine	1157.0 ± 23.2	1282.8 ± 29.7
Pyruvate	7147.3 ± 185.3	7092.3 ± 637.5
l-Serine	3066.4 ± 59.6	2931.0 ± 91.2
Spermidine	3490.7 ± 58.0	4203.9 ± 235.4
Succinate	2914.4 ± 148.7	2975.4 ± 198.2
l-Tryptophan	30.9 ± 8.7	12.4 ± 3.5
l-Tyrosine	331.5 ± 21.6	126.0 ± 3.6
UDP-Glucose	915.3 ± 35.6	1126.1 ± 46.5
Uracil	8.9 ± 0.4	6.5 ± 0.6
l-Valine	504.6 ± 30.2	143.4 ± 15.2
Xanthine	439.2 ± 18.0	111.6 ± 38.5

**Fig. 2:**
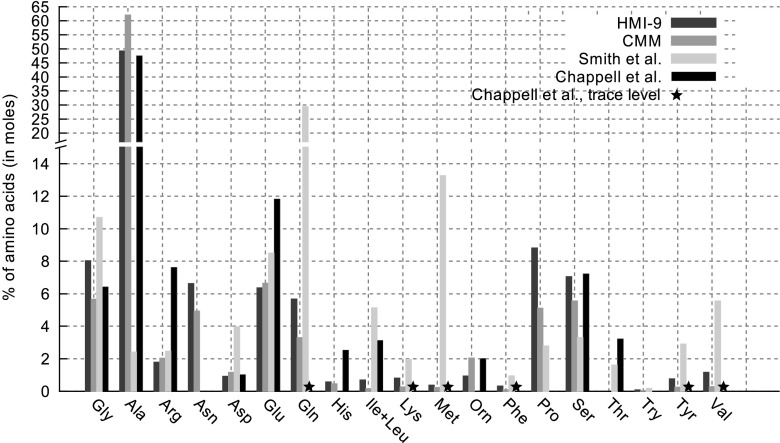
Comparison of the percentage of each amino acid in total intracellular amino acid concentrations in *T. brucei* to measurements reported in Chappell et al. (1972) and Smith et al. (2009)

For the validation of our LC–MS-based absolute quantification method, the measured intracellular concentrations of amino acids were compared to measurements in *Trypanosoma gambiense* and *T. brucei* reported in Chappell et al. (1972) and Smith et al. (2009). As can be seen in Fig. 2, overall, our measurements are matched relatively well with Chappell et al.: alanine is by far the most abundant free amino acid in both media as well as in Chappell et al. representing 49 % of the total free amino acid pools in HMI-9, 62 % in CMM and 47 % for Chappell et al. This is also the case in *Trypanosoma cruzi* (O’Daly et al. 1983) and Leishmania species (Simon et al. 1983; Vieira and Ioav Cabantchik 1995). Glutamate, serine and glycine represent more than 5 % of the free amino acids pools in all three series of measurements. The main difference between our measurements and Chappell et al. is the fraction of proline and asparagine (5–9 % each in CMM and HMI-9 but low in Chappell et al.) and the fraction of arginine (about 8 % in Chappell et al. about 2 % in CMM and HMI-9). However, differences in growth conditions, species and environments might have caused these variations, and this could also explain differences between the measurements reported by Smith et al. and the results of other studies.

### Metabolic flux measurement using extracellular concentrations

The measurement of extracellular metabolites excreted from cells into their growth media or consumed by the cells (Chong et al. 2009; Kaderbhai et al. 2003; Kim et al. 2010) can be connected to intracellular flux states (Mo et al. 2009). Therefore, quantitative analysis of extracellular metabolites in a growth medium can be employed for an understanding of cellular metabolism and the measurement of metabolic fluxes of many metabolites which can feed into quantitative computational models of metabolism.

**Fig. 3:**
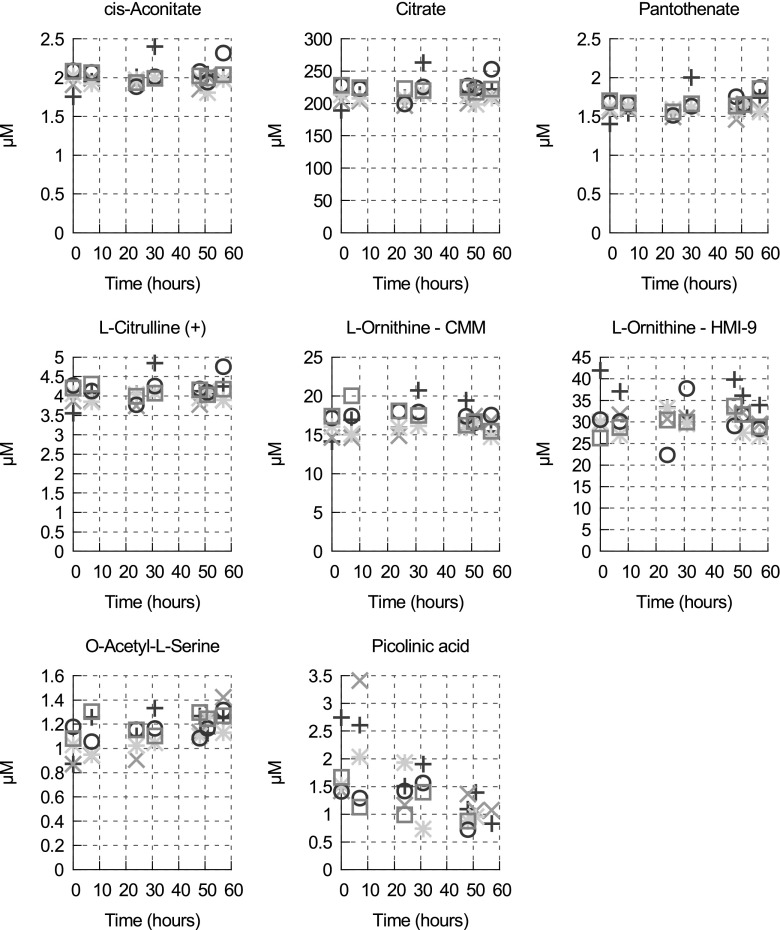
Variations of concentrations of the metabolites measured in CMM (unless otherwise specified) for which neither consumption nor production can be established over 57 h. The measurements where done in negative mode unless “+” is specified. Each type of symbol corresponds to a biological replicate of the time course

Extracellular metabolites were sampled with U-^13^C-labelled *E. coli* extract from the spent media at seven different time points (0, 7, 24, 31, 48, 51 and 57 h) and 32 metabolites were quantified by the IR-MS technique. It was confirmed in the previous study that no significant changes in any medium nutrient was observed in cell-free CMM incubated at 37 °C for 56 h (Creek et al. 2013). Extracellular concentrations from different time points are shown in Supplementary Table S4. Among these 32 metabolites, the concentrations of seven metabolites do not change over time in the medium or the variations are too small to allow flux quantification (see Fig. 3). It was previously shown that *T. brucei* lacks an arginase (Vincent et al. 2012), the enzyme that converts l-arginine to l-ornithine present notably in *Leishmania* (Roberts et al. 2004), and that *T. brucei* is capable of taking up l-ornithine from the medium (Vincent et al. 2012). Our results show that significant amounts of l-ornithine are not consumed in either CMM or HMI-9. However, assuming that cells require ornithine only to synthesise putrescine, spermidine and trypanothione, we can estimate the total concentration of ornithine required per cell as about 6.7 mM for cells grown in CMM and 5.2 mM in HMI-9 [using the concentrations of ornithine, putrescine and spermidine in Table 1 and the concentration of trypanothione (160 µM) measured by (Henderson et al. 1987)]. Given a cell volume of 5.89 µl/10^8^ cells (Ariyanayagam et al. 2005), the total quantity of ornithine required per cell is 3.96 × 10^−10^ µmol in CMM and 3.07 × 10^−10^ µmol in HMI-9 and considering the cell density after 57 h of culture (about 4.6 × 10^6^ cells/ml in CMM and 5.4 × 10^6^ cells/ml in HMI-9), the total concentration of ornithine in the medium would have decreased by 1.8 µM (1.8 × 10^−3^ µmol/ml) in CMM and 1.7 µM in HMI-9 after 57 h. This is clearly too small for us to see, given the variations of our measurement (see Fig. 3). This is also likely to be the case for pantothenate, which is required for coenzyme A biosynthesis.

**Fig. 4:**
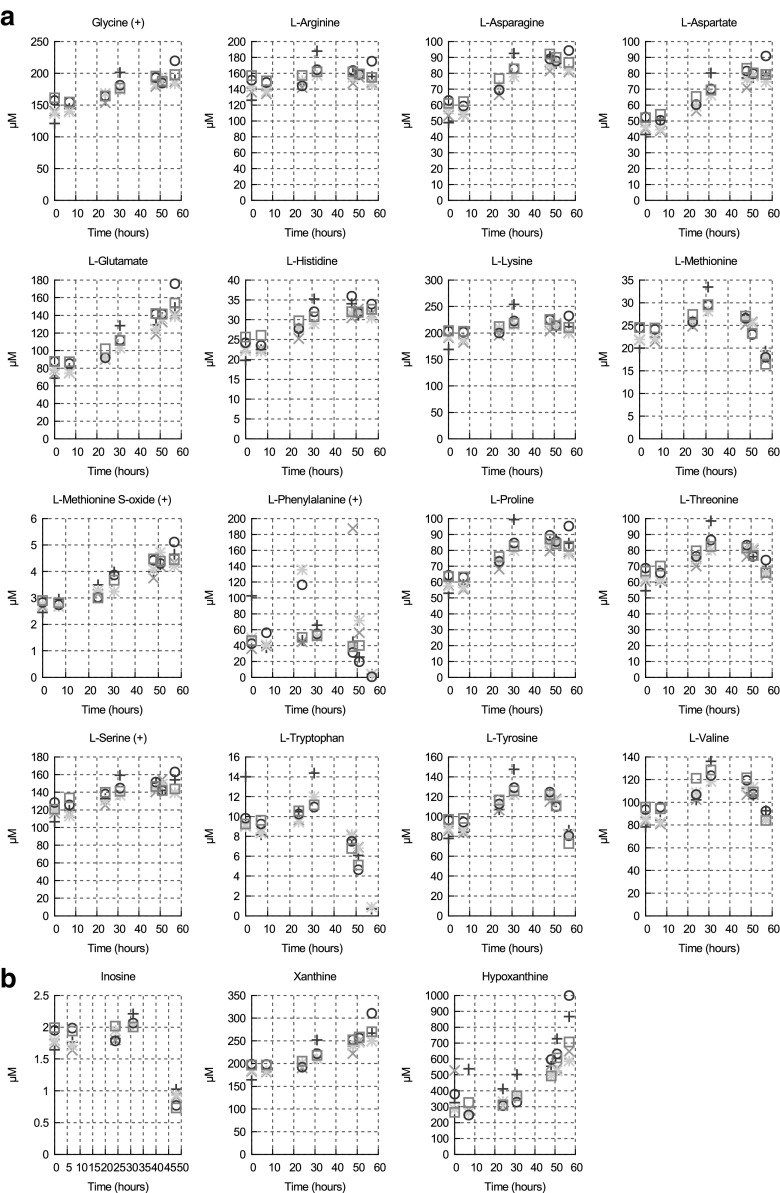
Concentrations of amino acids (**a**) and nucleoside/nucleotide (**b**) in CMM medium (negative mode unless otherwise specified) over 57 h. Each type of symbol corresponds to a biological replicate of the time course

For 19 metabolites, the time courses were not consistent with a constant production or consumption (see Fig. 4a, b). These metabolites are either amino acids (Fig. 4a) or nucleosides/nucleotides (Fig. 4b; Supplementary Figure S6). All of these compounds showed a similar pattern: over the first 31 h, while the cell number is still relatively low, the concentration of these metabolites increased; after 31 h they either decrease or continue to increase depending on the metabolite; in none of the cases do they show the exponential increase that would result from a constant production rate. It has been shown that *T. brucei* secretes numerous proteins, such as enzymes involved in nucleotide and protein hydrolysis (Geiger et al. 2010; Holzmuller et al. 2010; Troeberg et al. 1996). Therefore, the concentration increase in the first 31 h is most likely the result of amino acid and nucleotide released from proteins and nucleic acids respectively; with low cell density, the rate of production of these metabolites exceeds their consumption by the parasites. By 48 h, as the cell density increases and depending on the metabolite and its concentration: (i) the metabolite is now consumed more than it is produced, hence its disappearance from the medium, or (ii) it is produced more than it is consumed and it appears to increase (slowly) over time. Some metabolites are also produced by the parasite and excreted, and hence continue to increase, albeit at a decreasing rate.

**Fig. 5:**
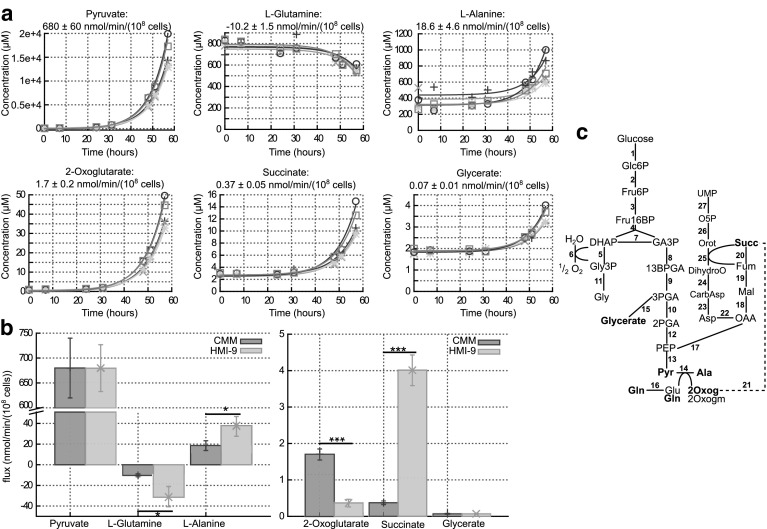
**a** Time-courses and fitted fluxes of constantly consumed or produced metabolites in CMM medium (negative mode). **b** Fluxes of constantly consumed or produced metabolites in both CMM and HMI-9 medium. Each type of symbol corresponds to a biological replicate of the time course. *p value < 0.05, ***p value < 0.001. **c** Simplified schematic of the part of bloodstream form *T. brucei* metabolism connected to the elements measured in (**a**), based on (Creek et al. 2015). *Glc6P* glucose 6-phosphate, *Fru6P* fructose 6-phosphate, *Fru16BP* fructose 1,6-bisphosphate, *DHAP* dihydroxyacetone phosphate, *GA3P* glyceraldehyde 3-phosphate, *Gly3P* glycerol 3-phosphate, *Gly* glycerol, *13BPGA* 1,3-bisphosphoglyceraldehyde, *3PGA* 3-phosphoglyceraldehyde, *2PGA* 2-phosphoglyceraldehyde, *PEP* phosphoenolpyruvate, *Pyr* pyruvate, *Ala* l-alanine, *Gln* l-glutamine, *Glu* -glutamate, *2Oxog* 2-oxoglutarate, *2Oxogm* 2-oxoglutaramate, *OAA* oxaloacetate, *Mal* malate, *Fum* fumarate, *Succ* succinate, *Asp* l-aspartate, *CarbAsp* carbamoyl-phosphate, *CarbAsp* *N*-carbamoyl-l-aspartate, *DihydroO* dihydroorotate, *Orot* orotate, *O5P* orotate 5-phosphate. *Reactions* *1* hexokinase, * 2* phosphoglucose isomerase, * 3* phosphofructokinase, * 4* aldolase, * 5* glycerol 3-phosphate dehydrogenase, * 6* glycerol 3-phosphate dehydrogenase + trypanosome alternative oxidase, * 7* triosephosphate isomerase, * 8* glyceraldehyde 3-phosphate dehydrogenase, *9* phosphoglycerate kinase, * 10* phosphoglycerate mutase, * 11* glycerol kinase, * 12* enolase, * 13* pyruvate kinase, * 14* alanine aminotransferase and glutamine-pyruvate transaminase (Marciano et al. 2009), * 15* the labelling pattern of glycerate in (Creek et al. 2015) shows that it is most likely produced from triose-phosphate from glycolysis. The exact reaction and enzyme is not known, * 16* glutaminase, * 17* phosphoenolpyruvate carboxykinase, * 18* malate dehydrogenase, * 19* fumarase, * 20* succinate dehydrogenase, * 21* possible pathway producing succinate from 2-oxoglutarate. Exact reactions to be determined in bloodstream form, * 22* aspartate aminotransferase, * 23* aspartate carbamoyltransferase, * 24* dihydroorotase, * 25* dihydroorotate dehydrogenase,* 26* orotate phosphoribosyltransferase, * 27* orotidine-5′-phosphate decarboxylase; part of this pathway is localised in the glycosomes, part is in the cytosol and part remains to be determined

Finally, six metabolites appear to be constantly produced or consumed and thus show an exponential increase or decrease over time (see Fig. 5a), allowing quantification of their production or consumption (see Sect. 2) when cells are grown in CMM and HMI-9 (see Fig. 5b). Glutamine is added to the medium both in CMM (1 mM) and HMI-9 (4 mM) and is consumed rapidly (compared to other amino acids); therefore, any small increase due to peptidase activity is not significant. Similarly, l-alanine is produced from pyruvate and excreted rapidly by the cells, hence any increase due to peptidase action in the medium is not significant and fluxes can be estimated. As expected, the pyruvate production flux is the highest flux measured. This flux is the same whether cells are grown in CMM or HMI-9 medium. The pyruvate flux we measured in HMI-9 is substantially higher than the flux reported by Haanstra et al. (2012). They measured about 370 nmol/(min 10^8^ cells) (calculated from the glucose consumption flux and the pyruvate production to glucose consumption ratio) whilst we measured 680 ± 47 nmol/(min 10^8^ cells) despite similar growth rates (see Supplementary Figure S7). A difference between experiments in which flux was measured here and the results of Haanstra et al. is the cell density at the beginning of the time-course, which might account for the measured differences.

In addition to pyruvate, l-alanine, 2-oxoglutarate, succinate and glycerate are constantly produced by the cells. The l-alanine produced has been shown to come primarily from pyruvate (Creek et al. 2015) via alanine aminotransferase (Ariyanayagam et al. 2005). Hence the glutamine consumed is probably used as an amino group donor, which would explain why in HMI-9 where the alanine production is higher, the glutamine consumption is also higher. Each glutamine can donate up to two amino groups and other amino acids can also be used as amino group donors, explaining why alanine production flux can exceed the glutamine consumption flux. The cells then excrete either glutamate (see Fig. 4a) or 2-oxoglutarate or possibly 2-oxoglutaramate (annotation of the latter based on exact mass only). Interestingly, the cells excrete more 2-oxoglutarate in CMM than in HMI-9 despite consuming less glutamine. Two hypotheses could explain such a discrepancy: (i) the cells excrete more glutamate or 2-oxoglutaramate in HMI-9, which would mean that in the presence of more glutamate (4 mM in HMI-9, 1 mM in CMM) the cells only use one of the two amino groups available in glutamine, (ii) the cells could further metabolise 2-oxoglutarate more in HMI-9. The latter hypothesis is further strengthened by the tenfold higher rate of production of succinate in HMI-9 as compared to CMM. Indeed, the succinate produced has been shown to come only in part from glucose, the rest most probably coming from amino acids (Creek et al. 2015). The small amount of glycerate produced comes from glucose (Creek et al. 2015) and is similar in both media.

It is worth noting that our measurements of the fluxes of succinate and alanine production are similar to those recently measured by Mazet et al. using NMR (Mazet et al. 2013) [1.2 nmol/(min 10^8^ cells) of succinate and 14 nmol/(min 10^8^ cells) of alanine], although the pyruvate production flux that they measured [130 nmol/(min 10^8^ cells) is significantly lower than that measured here or in Haanstra et al. (2012], probably due to stressful culture conditions in Mazet et al. (2013) (5 h in phosphate-buffered saline solution with added glucose but no other nutrients).

### Metabolites detected in the medium that could not be absolutely quantified

Supplementary Figure 6 contains the time courses of metabolites identified in the medium (exact mass and retention time matching with their standards) that could not be quantified, either because the metabolite is absent in *E. coli* or because the concentration is beyond the dynamic range of our calibration curve (either in the medium or in *E. coli*). Among them, 11 compounds (l-cysteine, phenylpyruvate, imidazole-4-acetate, hydroxyglutarate, glucuronolactone, glucono-1,4-lactone, nicotinic acid, thymine and orotate) seem to be constantly produced or consumed. l-cysteine is known to be consumed in large quantities and is essential to *T. brucei* (Creek et al. 2013; Duszenko et al. 1992). Phenylpyruvate is the keto-acid produced from l-phenylalanine. Similarly, imidazole 4-acetate might be a degradation product or fragment of imidazole 4-pyruvate, the keto-acid produced from l-histidine and hydroxyglutarate is the reduced form of 2-oxoglutarate, the keto-acid produced from l-glutamate. Indeed, it has long been known that *T. brucei* excretes numerous of these keto-acids (Berger et al. 1996; Creek et al. 2013; Stibbs and Seed 1973, 1975) that are produced via promiscuous aminotransferases (Ariyanayagam et al. 2005; Marciano et al. 2008).

Nicotinic acid is consumed by the cells where it is used in the production of nicotinamide-based cofactors e.g., NAD(H) and NADP(H). While it cannot be excluded with certainty that the detected compound is actually isonicotinic acid, based on exact mass and retention time similarity to the nicotinic acid standard, it is here putatively identified as nicotinic acid, as isonicotinic acid is not involved in any pathways or reactions documented for trypanosomes, in contrast to nicotinate which is a part of the NAD salvage pathway.

Thymine and orotate can be produced via degradation of nucleotides. They might therefore be expected to be acquired from medium as we see after 31 h for other nucleotides. However, since trypanosomes are capable of synthesising thymine and orotate from glucose (Creek et al. 2015) there is no observed decrease in the medium. Indeed, for thymine no uptake could be observed (Gudin et al. 2006).

The origin of the increased concentration of carbohydrate lactones is assumed to be the oxidation of glucose in the medium over the time course.

## Conclusion

We report a practical methodology using an isotope ratio-based mass spectrometry technique for the measurement of intra- and extra-cellular concentrations of 43 metabolites in *T. brucei* and its spent medium. This study also demonstrates that the technique is a useful tool for the estimation of metabolic fluxes of the metabolites, providing an accurate and unbiased method for the analysis of intracellular metabolism. The computed metabolic fluxes will now allow us to identify the most plausible metabolic network topology and drive the extension of the existing computational models to a more comprehensive representation of the metabolic capacity of the parasite (Achcar et al. 2014; Bakker et al. 2010). Importantly, this absolute quantitative technique can be easily transferred to other organisms that cannot be grown on glucose as the sole carbon source, including human cells.

## Electronic supplementary material

Below is the link to the electronic supplementary material.

Supplementary material 1 (PDF 91 kb). Supplementary file A – PDF of R script of mzMatch and mzMatch-ISO for data processing

Supplementary material 2 (PDF 94 kb). Supplementary table S2 – PDF of the list of calibration standards with 5 groups

Supplementary material 3 (PDF 111 kb). Supplementary table S3 – PDF of the complete list of the common putative metabolites in *E. coli* and *T. brucei* extracts

Supplementary material 4 (PDF 91 kb). Supplementary table S4 – PDF of concentrations of extracellular metabolites from spent CMM media where *T. brucei* blood stream forms were grown at different time points (0, 7, 24, 31, 48, 51 and 57 h)

Supplementary material 5 (PDF 155 kb). Supplementary figure S6 – PDF of concentrations of metabolites from the time courses identified in CMM medium that could not be quantified. Each colour corresponds to a biological replicate of the time course

Supplementary material 6 (PDF 123 kb). Supplementary figure S7 – PDF of growths of 5 independent cultures of *T. brucei* bloodstream form grown in CMM (+) and HMI-9 (×). Open and closed circle: growth of two independent cultures of *T. brucei* bloodstream form of Haanstra et al. (2012)
