# Supplementary material for: LC–MS-based absolute metabolite quantification: application to metabolic flux measurement in trypanosomes
Source: Metabolomics. 2015 Jul 9;11(6):1721–32. doi: 10.1007/s11306-015-0827-2 (PMC4605981; doi:10.1007/s11306-015-0827-2)
Supplement: Supplementary file 1 — Supplementary material 1 (PDF 91 kb). Supplementary file A – PDF of R script of mzMatch and mzMatch-ISO for data processing [file 11306_2015_827_MOESM1_ESM.pdf]

# SUPPLEMENTARY INFORMATION

# LC-MS-based absolute metabolite quantification: Application to metabolic flux measurement in trypanosomes

Dong-Hyun Kim[1,2], Fiona Achcar[1], Rainer Breitling[3], Karl E. Burgess[4], Michael P. Barrett[1,4*]

[1]Wellcome Trust Centre for Molecular Parasitology, Institute of Infection, Immunity and Inflammation, College of Medical Veterinary and Life Sciences, University of Glasgow, G12 8TA, UK

[2]Current: Centre for Analytical Bioscience, School of Pharmacy, University of Nottingham, University Park, Nottingham, NG7 2RD, UK

[3]Manchester Centre of Synthetic Biology for Fine and Speciality Chemicals, Manchester Institute of Biotechnology, Faculty of Life Sciences, University of Manchester, Manchester, M1 7DN, UK.

[4]Glasgow Polyomics, Wolfson Wohl Cancer Research Centre, College of Medical Veterinary & Life Sciences, University of Glasgow, G61 1QH, UK

*Address for Correspondence:

M.P. Barrett, Wellcome Trust Centre for Molecular Parasitology, Institute of Infection, Immunity and Inflammation, College of Medical Veterinary and Life Sciences, University of Glasgow, G12 8TA, UK
E-mail: Michael.Barrett@glasgow.ac.uk
Tel: +44 (0) 141 330 6904. Fax: +44 (0) 141 330 4077

```
## load required packages

library(mzmatch.R)
mzmatch.init(15000)

##Functions
xsetFunction <- function (i)
{
	require (mzmatch.R)
	mzmatch.init ()
	PeakML.xcms.write.SingleMeasurement(xsets[[i]], filename=outputfilenames[i],ionisation='detect',addscans=0,ppm=5,writeRejected=FALSE,ApodisationFilter=TRUE)
}


## Organising experimental data set

setwd ('INPUT_DIR')

mzXMLfiles.fullnames <- dir(full.names=TRUE,pattern='\\.mzXML$',recursive=TRUE)
mzXMLfiles.shortnames <- dir(full.names=FALSE,pattern='\\.mzXML$',recursive=TRUE)
outputfilenames <- paste(sub('.mzXML', '', mzXMLfiles.fullnames), '.peakml', sep='')

# Extracting peaks with centWave algorithm from XCMS

xseto <- xcmsSet(mzXMLfiles.fullnames, method='centWave', ppm=2, peakwidth=c(10,100), snthresh=5, prefilter=c(3,1000), integrate=1, mzdiff=0.01, verbose.columns=TRUE, fitgauss=FALSE,nSlaves=4)

# RT correction to disable for isotope labelling studies
if(T==T){
	#xset2<-retcor(xseto, method='obiwarp', profStep=0.01, center=1)
	xset2<-retcor(xseto, method='obiwarp', profStep=0.01)
	print('RT correction done\n')
}else{
	xset2 <- xseto
}

## Write out peaks as single peakml files
xsets <- split (xset2,xset2@filepaths)

cl <- makeCluster (4)
clusterExport (cl,list=c('xsetFunction','xsets','outputfilenames','mzXMLfiles.fullnames'))
system.time(clusterApply(cl,1:length(mzXMLfiles.fullnames),xsetFunction))
stopCluster(cl)

MainClasses <- dir ()
```

```
if(T){
          ## Combine biological replicates
          dir.create ('combined')
          NoReplicates=c()
          for (i in 1:length(MainClasses))
          {
            FILESf <- dir(MainClasses[i],full.names=TRUE,pattern='\\.peakml$',recursive=TRUE)
            OUTPUTf <- paste('combined/',MainClasses[i],'.peakml',sep='')
            mzmatch.ipeak.Combine(
                i=paste(FILESf, collapse=','), v=T, rtwindow=60,
                o=OUTPUTf, combination='set',
                ppm=5, label=paste(MainClasses[i], sep='')
              )
            NoReplicates=append(NoReplicates, length(FILESf[i])==1)
          }

          print('Check one of the file in combined/ if pics are correctly aligned. If not run rt correction')

          ## RSD filtering
          dir.create ('combined_RSD_filtered')
          dir.create ('combined_RSD_rejected')
          FILESf <- dir ('combined',full.names=TRUE)
          FILESs <- dir ('combined',pattern='\\.peakml$')
          for (i in 1:length(FILESf))
          {
                    outputf=paste('combined_RSD_filtered/', FILESs[i], sep='')
                    if(NoReplicates[i]){
                              file.copy(FILESf[i], outputf)
                    }else{
                              mzmatch.ipeak.filter.RSDFilter(
                                        i=FILESf[i],
                                        o=outputf,
                                        rejected=paste('combined_RSD_rejected/',FILESs[i],sep=''),
                                        rsd=0.3, v=T
                              )
                    }
          }

          ## Combine by conditions
          INPUTDIR <- 'combined_RSD_filtered'
          FILESf <- dir (INPUTDIR,full.names=TRUE,pattern='\\.peakml$')
          mzmatch.ipeak.Combine(
              i=paste(FILESf,collapse=','), v=T, rtwindow=30,
              o='final_combined.peakml', combination='set', ppm=5
          )
```

```
}else{
            # Combining
            FILESf <- dir(full.names=TRUE, pattern='\\.peakml$')
            mzmatch.ipeak.Combine(i=paste(FILESf, collapse=','), v=T, rtwindow=30, o='final_combined.peakml', combination='set', ppm=5)

}


## Filtering
mzmatch.ipeak.filter.NoiseFilter (i='final_combined.peakml',o='combined_nf.peakml',v=T, codadw=0.5)
mzmatch.ipeak.filter.SimpleFilter(i='combined_nf.peakml', o='combined_sf1.peakml', mindetections=1, v=T)
mzmatch.ipeak.filter.SimpleFilter(i='combined_sf1.peakml', o='combined_sf2.peakml', minintensity=1000, v=T)
#mzmatch.ipeak.filter.SimpleFilter(i='combined_sf1.peakml', o='combined_sf2.peakml', minintensity=10000, v=T)

## Gap Filler
PeakML.GapFiller(filename = 'combined_sf2.peakml', ionisation = 'detect', outputfile = 'combined_gapfilled.peakml', ppm = 0, rtwin = 0, fillAll=TRUE,Rawpath='INPUT_DIR')

# Remove repeating group IDs
PeakML.FilterPeakGroups(filename = 'combined_gapfilled.peakml', ionisation = 'detect', outputfile = 'combined_cleaned.peakml', ppm = 5, rtwin = 15, Rawpath='INPUT_DIR')

## Match related peaks

mzmatch.ipeak.sort.RelatedPeaks(
   i='combined_cleaned.peakml', v=T,
   o='final_combined_related.peakml',
   basepeaks='final_combined_basepeaks.peakml',
   ppm=5, rtwindow=30,
   JHeapSize=15000
  )

## Identify peaks from data bases

DBS <- dir( paste(.find.package('mzmatch.R'), '/dbs', sep=''), full.names=TRUE)

DBS <- paste(DBS[c(1,2,3,4,7,8,9,11,13)],collapse=',')
mzmatch.ipeak.util.Identify(
    i='final_combined_related.peakml', v=T,
    o='final_combined_related_identified.peakml',
    ppm=5, databases=DBS,
   JHeapSize=15000
  )
mzmatch.ipeak.util.Identify(
   i='final_combined_basepeaks.peakml', v=T,
   o='final_combined_basepeaks_identified.peakml',
```

```
    ppm=5, databases=DBS,
    JHeapSize=15000
  )

PeakML.Isotope.TargettedIsotopes(
                    baseDir='INPUT_DIR',
                    molFormulaFile='list_of_metabolites_file.tab',
                    outFileName='output_file',
                    mzXMLSrc='INPUT_DIR',
                    useArea =TRUE,
                    peakMLFile='final_combined_related_identified.peakml',
                    label=1,
                    fillGaps='ALLPEAKS',
)
```
