# Supplementary material for: LC–MS-based absolute metabolite quantification: application to metabolic flux measurement in trypanosomes
Source: Metabolomics. 2015 Jul 9;11(6):1721–32. doi: 10.1007/s11306-015-0827-2 (PMC4605981; doi:10.1007/s11306-015-0827-2)
Supplement: Supplementary file 2 — Supplementary material 2 (PDF 94 kb). Supplementary table S2 – PDF of the list of calibration standards with 5 groups [file 11306_2015_827_MOESM2_ESM.pdf]

SUPPLEMENTARY INFORMATION

LC-MS-based absolute metabolite quantification: Application to metabolic flux measurement in trypanosomes

Dong-Hyun Kim[1,2], Fiona Achcar[1], Rainer Breitling[3], Karl E. Burgess[4], Michael P. Barrett[1,4*]

[1]Wellcome Trust Centre for Molecular Parasitology, Institute of Infection, Immunity and Inflammation, College of Medical Veterinary and Life Sciences, University of Glasgow, G12 8TA, UK

[2]Current: Centre for Analytical Bioscience, School of Pharmacy, University of Nottingham, University Park, Nottingham, NG7 2RD, UK

[3]Manchester Centre of Synthetic Biology for Fine and Speciality Chemicals, Manchester Institute of Biotechnology, Faculty of Life Sciences, University of Manchester, Manchester, M1 7DN, UK.

[4]Glasgow Polyomics, Wolfson Wohl Cancer Research Centre, College of Medical Veterinary & Life Sciences, University of Glasgow, G61 1QH, UK

*Address for Correspondence:

M.P. Barrett, Wellcome Trust Centre for Molecular Parasitology, Institute of Infection, Immunity and Inflammation, College of Medical Veterinary and Life Sciences, University of Glasgow, G12 8TA, UK
E-mail: Michael.Barrett@glasgow.ac.uk
Tel: +44 (0) 141 330 6904. Fax: +44 (0) 141 330 4077

Group 1	Mass	Group 2	Mass	Group 3	Mass
Glycine	75.0320	Imidazole	68.0374	Methylguanidine	73.0640
Pyruvate	88.0161	methylglyoxal	72.0211	L-Alanine	89.0477
Putrescine	88.1000	Glyoxylate	74.0004	4-Aminobutanoate	103.0633
beta-Alanine	89.0477	glycolate	76.0160	Choline	104.1070
Glycerol	92.0473	glyceraldehyde	90.0317	cytosine	111.0433
L-Serine	105.0426	2-oxobutanoate	102.0317	Uracil	112.0273
L-Proline	115.0633	Malonate	104.0110	L-homoserine	119.0582
L-Valine	117.0790	(R)-3-Hydroxybutanoate	104.0474	Nicotinamide	122.0480
L-2,4-Diaminobutanoate	118.0742	Fumarate	116.0110	Picolinic acid	123.0320
L-Threonine	119.0582	Methylmalonate	118.0266	thymine	126.0429
Nicotinate	123.0321	D-Erythrose	120.0423	5-Oxoproline	129.0426
Taurine	125.0147	Oxaloacetate	132.0059	agmatine	130.1218
Imidazole-4-acetate	126.0429	Deoxyribose	134.0579	L-isoleucine	131.0946
trans-4-Hydroxy-L-proline	131.0582	Adenine	135.0545	L-Ornithine	132.0899
L-Leucine	131.0946	L-Glutamate	147.0532	Methylcysteine	135.0354
L-Asparagine	132.0535	(R)-2-Hydroxyglutarate	148.0372	L-Carnitine	162.1125
L-Aspartate	133.0295	Xanthine	152.0334	Pyridoxal	167.0582
4-Aminobenzoate	137.0477	3-Hydroxyphenylacetate	152.0473	N(pi)-Methyl-L-histidine	169.0851
Ethanolamine phosphate	141.0191	Orotate	156.0171	N-Acetylornithine	174.1004
Spermidine	145.1579	L-Rhamnose	164.0685	L-Tyrosine	181.0739
L-Glutamine	146.0691	Phosphoenolpyruvate	167.9824	Choline phosphate	184.0739
L-Lysine	146.1055	cis-Aconitate	174.0164	Spermine	202.2157
L-Methionine	149.0511	D-glucose	180.0634	L-Kynurenine	208.0848
Guanine	151.0494	D-Gluconic acid	196.0583	Pantothenate	219.1107
2-Phenylglycine	151.0633	2-Methylcitrate	206.0426	L-Cystathionine	222.0674
L-Histidine	155.0695	D-Galactarate	210.0376	Deoxyadenosine	251.1018
Pyridoxine	169.0739	D-ribose 5-phosphate	230.0192	D-Glucosamine 6-phosphate	259.0457
L-Dehydroascorbate	174.0164	L-Cystine	240.0238	Adenosine	267.0967
L-Arginine	174.1117	Uridine	244.0695	Guanosine	283.0917
L-Citrulline	175.0957	Pyridoxal phosphate	247.0246	dCMP	307.0569
D-Glucosamine	179.0794	D-glucose 6-phosphate	260.0297	5'-Methylthioadenosine	313.0845
L-Phenylalanine	179.0946	2,3-Bisphospho-D-glycerate	265.9593	dAMP	331.0682
N2-Acetyl-L-lysine	188.1161	6-Phospho-D-gluconate	276.0246	AMP	347.0631
Selenomethionine	196.9955	UMP	324.0359	Folate	441.1397
L-Tryptophan	204.0899	3',5'-Cyclic AMP	329.0525	NAD+	664.1164
N-Acetyl-D-glucosamine	221.0899	D-Fructose 1,6-bisphosphate	339.9961	Trypanothione disulfide	721.2888
Deoxyuridine	228.0746	Maltose	342.1162	FAD	785.1571
Thymidine	242.0903	IMP	348.0471		
Cytidine	243.0855	CMP	375.9349		
sn-glycero-3-Phosphocholine	258.1101	Thiamin diphosphate	425.0450		
Thiamin	265.1123	ADP	427.0294		
Inosine	268.0808	IDP	428.0134		
dGMP	347.0631	CoA	767.1152		
S-Adenosyl-L-methionine	399.1451				

Group 4	Mass	Group 5	Mass
1,3-Diaminopropane	74.0844	(R)-Lactate	90.0317
Oxalate	89.9953	Glycerate	106.0266
Succinate semialdehyde	102.0317	Maleic acid	116.0110
Succinate	118.0266	3-Methyl-2-oxobutanoic acid	116.0473
D-Threose	120.0423	L-cysteine	121.0197
(S)-Malate	134.0215	Mesaconate	130.0266
Hypoxanthine	136.0385	5-Aminolevulinate	131.0582
2-Oxoglutarate	146.0215	L-Homocysteine	135.0354
D-Ribose	150.0528	Ectoine	142.0742
Allantoin	158.0440	Acetylcholine	146.1176
Phenylpyruvate	164.0473	O-Acetyl-L-serine	147.0532
2-Deoxy-D-glucose	164.0685	(S)-Dihydroorotate	158.0328
DL-Glyceraldehyde 3-phosphate	169.9980	acetylcysteine	163.0303
sn-Glycerol 3-phosphate	172.0137	L-Methionine S-oxide	165.0460
D-Glucuronolactone	176.0321	ascorbate	176.0321
3-(4-Hydroxyphenyl)pyruvate	180.0423	D-Glucono-1,4-lactone	178.0477
3-Phospho-D-glycerate	185.9929	D-Fructose	180.0634
N-Acetylglutamine	188.0797	2-phospho-D-glycerate	185.9929
Citrate	192.0270	N6-Acetyl-L-Lysine	188.1161
Phenylacetylglycine	193.0739	N-acetyl-L-glutamate	189.0637
D-Erythrose 4-phosphate	200.0086	O-Acetylcarnitine	204.1230
Orotidine	288.0594	Biotin	244.0882
dUMP	308.0410	gamma-L-Glutamyl-L-cysteine	250.0623
2'-Deoxyinosine 5'-phosphate	332.0522	D-Fructose 6-phosphate	260.0297
GMP	363.0580	Deoxyguanosine	267.0967
S-Adenosyl-L-homocysteine	384.1216	Homocystine	268.0551
dGDP	427.0294	Glutathione	307.0838
GDP	443.0243	UDP	404.0022
FMN	456.1046	Adenosine 2',5'-bisphosphate	427.0294
CTP	482.9845	UTP	483.9685
ATP	506.9958	NADH	665.1248
GTP	522.9907	trypanothione	723.3044
UDP-glucose	566.0550	NADP	743.0754
NADP+	743.0754	Acetyl-CoA	809.1258
