# Supplementary material for: LC–MS-based absolute metabolite quantification: application to metabolic flux measurement in trypanosomes
Source: Metabolomics. 2015 Jul 9;11(6):1721–32. doi: 10.1007/s11306-015-0827-2 (PMC4605981; doi:10.1007/s11306-015-0827-2)
Supplement: Supplementary file 3 — Supplementary material 3 (PDF 111 kb). Supplementary table S3 – PDF of the complete list of the common putative metabolites in E. coli and T. brucei extracts [file 11306_2015_827_MOESM3_ESM.pdf]

SUPPLEMENTARY INFORMATION

LC-MS-based absolute metabolite quantification: Application to metabolic flux measurement in trypanosomes

Dong-Hyun Kim[1,2], Fiona Achcar[1], Rainer Breitling[3], Karl E. Burgess[4], Michael P. Barrett[1,4*]

[1]Wellcome Trust Centre for Molecular Parasitology, Institute of Infection, Immunity and Inflammation, College of Medical Veterinary and Life Sciences, University of Glasgow, G12 8TA, UK

[2]Current: Centre for Analytical Bioscience, School of Pharmacy, University of Nottingham, University Park, Nottingham, NG7 2RD, UK

[3]Manchester Centre of Synthetic Biology for Fine and Speciality Chemicals, Manchester Institute of Biotechnology, Faculty of Life Sciences, University of Manchester, Manchester, M1 7DN, UK.

[4]Glasgow Polyomics, Wolfson Wohl Cancer Research Centre, College of Medical Veterinary & Life Sciences, University of Glasgow, G61 1QH, UK

*Address for Correspondence:

M.P. Barrett, Wellcome Trust Centre for Molecular Parasitology, Institute of Infection, Immunity and Inflammation, College of Medical Veterinary and Life Sciences, University of Glasgow, G12 8TA, UK
E-mail: Michael.Barrett@glasgow.ac.uk
Tel: +44 (0) 141 330 6904. Fax: +44 (0) 141 330 4077

	Mass	RT	Formula	Putative metabolite	Map	PubChem ID
1	189.0822	8.20	C8H15NO2S	Prenyl-L-cysteine	Amino Acid Metabolism	PubChem:25200435
2	121.0196	15.67	C3H7NO2S	L-Cysteine	Amino Acid Metabolism	PubChem:5862
3	297.0897	8.38	C11H15N5O3S	5'-Methylthioadenosine	Amino Acid Metabolism	PubChem:439176
4	103.0633	13.98	C4H9NO2	N,N-Dimethylglycine	Amino Acid Metabolism	PubChem:673
5	139.0745	12.38	C6H9N3O	L-Histidinal	Amino Acid Metabolism	PubChem:57339283
6	88.1000	27.15	C4H12N2	Putrescine	Amino Acid Metabolism	PubChem:1045
7	117.0539	12.02	C3H7N3O2	Guanidinoacetate	Amino Acid Metabolism	PubChem:763
8	118.0742	22.72	C4H10N2O2	L-2,4-Diaminobutanoate	Amino Acid Metabolism	PubChem:6971272
9	161.0689	12.73	C6H11NO4	O-Acetyl-L-homoserine	Amino Acid Metabolism	PubChem:25244888
10	145.1579	37.43	C7H19N3	Spermidine	Amino Acid Metabolism	PubChem:1102
11	73.0528	10.28	C3H7NO	Aminoacetone	Amino Acid Metabolism	PubChem:215
12	75.0320	17.13	C2H5NO2	Glycine	Amino Acid Metabolism	PubChem:750
13	89.0476	16.29	C3H7NO2	L-Alanine	Amino Acid Metabolism	PubChem:5950
14	102.0317	17.37	C4H6O3	2-Oxobutanoate	Amino Acid Metabolism	PubChem:3593277
15	103.0633	16.88	C4H9NO2	4-Aminobutanoate	Amino Acid Metabolism	PubChem:5460232
16	103.0997	19.94	C5H13NO	Choline	Amino Acid Metabolism	PubChem:305
17	105.0426	17.30	C3H7NO3	L-Serine	Amino Acid Metabolism	PubChem:5951
18	111.9926	16.33	CH5O4P	Hydroxymethylphosphonate	Amino Acid Metabolism	PubChem:21680357
19	115.0633	13.89	C5H9NO2	L-Proline	Amino Acid Metabolism	PubChem:145742
20	116.0473	16.54	C5H8O3	3-Methyl-2-oxobutanoic acid	Amino Acid Metabolism	PubChem:49
21	117.0790	12.07	C5H11NO2	Betaine	Amino Acid Metabolism	PubChem:247
22	117.0790	13.22	C5H11NO2	L-Valine	Amino Acid Metabolism	PubChem:6287
23	119.0583	15.97	C4H9NO3	L-Threonine	Amino Acid Metabolism	PubChem:6288
24	126.1156	13.97	C7H14N2	1-5-diazabicyclononane	Amino Acid Metabolism	PubChem:25203381
25	127.0633	7.32	C6H9NO2	2,3,4,5-Tetrahydropyridine-2-carboxylate	Amino Acid Metabolism	PubChem:3765
26	128.0950	13.38	C6H12N2O	L-Lysine 1,6-lactam	Amino Acid Metabolism	PubChem:440599
27	129.0426	11.72	C5H7NO3	L-1-Pyrroline-3-hydroxy-5-carboxylate	Amino Acid Metabolism	PubChem:11966267
28	129.0790	26.03	C6H11NO2	L-Pipecolate	Amino Acid Metabolism	PubChem:6931663
29	129.0790	7.01	C6H11NO2	N4-Acetylaminobutanal	Amino Acid Metabolism	PubChem:440850
30	129.0902	27.57	C5H11N3O	4-Guanidinobutanal	Amino Acid Metabolism	PubChem:25200663
31	130.1106	21.40	C6H14N2O	N-Acetylputrescine	Amino Acid Metabolism	PubChem:122356

	Mass	RT	Formula	Putative metabolite	Map	PubChem ID
32	131.0582	15.43	C5H9NO3	L-Glutamate 5-semialdehyde	Amino Acid Metabolism	PubChem:193305
33	131.0946	11.66	C6H13NO2	L-Leucine	Amino Acid Metabolism	PubChem:6106
34	132.0423	7.01	C5H8O4	(S)-2-Acetolactate	Amino Acid Metabolism	PubChem:440878
35	132.0898	24.34	C5H12N2O2	L-Ornithine	Amino Acid Metabolism	PubChem:6262
36	133.0374	16.76	C4H7NO4	L-Aspartate	Amino Acid Metabolism	PubChem:5960
37	135.0354	14.41	C4H9NO2S	L-Homocysteine	Amino Acid Metabolism	PubChem:91552
38	142.0741	14.27	C6H10N2O2	Ectoine	Amino Acid Metabolism	PubChem:126041
39	144.1262	20.15	C7H16N2O	1-(3-aminopropyl)-4-aminobutanal	Amino Acid Metabolism	PubChem:19913541
40	145.0375	12.02	C5H7NO4	2-Oxoglutaramate	Amino Acid Metabolism	PubChem:48
41	145.0853	16.57	C5H11N3O2	4-Guanidinobutanoate	Amino Acid Metabolism	PubChem:500
42	145.1102	10.77	C7H15NO2	3-Dehydroxycarnitine	Amino Acid Metabolism	PubChem:725
43	146.0693	16.54	C5H10N2O3	L-Glutamine	Amino Acid Metabolism	PubChem:5961
44	146.1055	25.92	C6H14N2O2	L-Lysine	Amino Acid Metabolism	PubChem:5962
45	147.0532	16.47	C5H9NO4	L-Glutamate	Amino Acid Metabolism	PubChem:33032
46	147.0532	12.15	C5H9NO4	O-Acetyl-L-serine	Amino Acid Metabolism	PubChem:99478
47	148.0371	16.95	C5H8O5	(R)-2-Hydroxyglutarate	Amino Acid Metabolism	PubChem:5460200
48	149.0511	12.70	C5H11NO2S	L-Methionine	Amino Acid Metabolism	PubChem:6137
49	154.0377	11.74	C6H6N2O3	Imidazol-5-yl-pyruvate	Amino Acid Metabolism	PubChem:23615390
50	155.0695	16.51	C6H9N3O2	L-Histidine	Amino Acid Metabolism	PubChem:6274
51	156.0536	12.85	C6H8N2O3	4-Imidazolone-5-propanoate	Amino Acid Metabolism	PubChem:128
52	159.0895	6.97	C7H13NO3	5-Acetamidopentanoate	Amino Acid Metabolism	PubChem:439903
53	160.0370	16.50	C6H8O5	2-Oxoadipate	Amino Acid Metabolism	PubChem:71
54	160.0848	12.86	C6H12N2O3	D-Alanyl-D-alanine	Amino Acid Metabolism	PubChem:5460362
55	160.1211	23.63	C7H16N2O2	N6-Methyl-L-lysine	Amino Acid Metabolism	PubChem:164795
56	161.1051	14.52	C7H15NO3	L-Carnitine	Amino Acid Metabolism	PubChem:10917
57	164.0474	4.49	C9H8O3	Phenylpyruvate	Amino Acid Metabolism	PubChem:997
58	165.0460	15.00	C5H11NO3S	L-Methionine S-oxide	Amino Acid Metabolism	PubChem:847
59	165.0790	11.14	C9H11NO2	L-Phenylalanine	Amino Acid Metabolism	PubChem:6140
60	166.0630	4.64	C9H10O3	3-Methoxy-4-hydroxyphenylacetaldehyde	Amino Acid Metabolism	PubChem:151276
61	166.0630	6.83	C9H10O3	3-(3-Hydroxy-phenyl)-propanoic acid	Amino Acid Metabolism	PubChem:91
62	173.0687	14.76	C7H11NO4	N-Acetyl-L-glutamate 5-semialdehyde	Amino Acid Metabolism	PubChem:192878
63	173.0800	15.52	C6H11N3O3	5-Guanidino-2-oxopentanoate	Amino Acid Metabolism	PubChem:25244978

	Mass	RT	Formula	Putative metabolite	Map	PubChem ID
64	174.1003	17.24	C7H14N2O3	N-Acetylornithine	Amino Acid Metabolism	PubChem:439232
65	174.1117	27.54	C6H14N4O2	L-Arginine	Amino Acid Metabolism	PubChem:6322
66	175.0956	16.89	C6H13N3O3	L-Citrulline	Amino Acid Metabolism	PubChem:9750
67	176.0684	14.18	C7H12O5	(2S)-2-Isopropylmalate	Amino Acid Metabolism	PubChem:5280523
68	180.0423	7.93	C9H8O4	3-(4-Hydroxyphenyl)pyruvate	Amino Acid Metabolism	PubChem:979
69	181.0740	14.31	C9H11NO3	L-Tyrosine	Amino Acid Metabolism	PubChem:6057
70	182.0580	9.73	C9H10O4	3-(4-Hydroxyphenyl)lactate	Amino Acid Metabolism	PubChem:9378
71	188.1162	16.53	C8H16N2O3	N6-Acetyl-L-lysine	Amino Acid Metabolism	PubChem:92832
72	188.1525	23.43	C9H20N2O2	N6,N6,N6-Trimethyl-L-lysine	Amino Acid Metabolism	PubChem:6992003
73	192.0634	16.36	C7H12O6	Quinate	Amino Acid Metabolism	PubChem:6508
74	195.0892	4.86	C10H13NO3	L-Tyrosine methyl ester	Amino Acid Metabolism	PubChem:70652
75	203.0795	14.87	C8H13NO5	N2-Acetyl-L-aminoadipate	Amino Acid Metabolism	PubChem:443992
76	204.0898	12.64	C11H12N2O2	L-Tryptophan	Amino Acid Metabolism	PubChem:6305
77	216.1109	13.41	C9H16N2O4	gamma-Glutamyl-gamma-aminobutyraldehyde	Amino Acid Metabolism	PubChem:25246104
78	219.1107	9.50	C9H17NO5	Pantothenate	Amino Acid Metabolism	PubChem:6613
79	232.1059	17.34	C9H16N2O5	N2-Succinyl-L-ornithine	Amino Acid Metabolism	PubChem:127370
80	247.0692	18.84	C9H13NO7	N-Succinyl-L-glutamate	Amino Acid Metabolism	PubChem:440847
81	274.1277	17.19	C10H18N4O5	N2-Succinyl-L-arginine	Amino Acid Metabolism	PubChem:439968
82	290.1112	17.96	C11H18N2O7	N-Succinyl-LL-2,6-diaminoheptanedioate	Amino Acid Metabolism	PubChem:
83	307.0839	16.16	C10H17N3O6S	Glutathione	Amino Acid Metabolism	PubChem:124886
84	132.0535	17.75	C4H8N2O3	L-Asparagine	Amino Acid Metabolism	PubChem:6267
85	194.0804	10.21	C8H10N4O2	Caffeine	Biosynthesis of Secondary Metabolites	PubChem:2519
86	72.0576	6.13	C4H8O	Butanal	Carbohydrate Metabolism	PubChem:261
87	134.0579	9.64	C5H10O4	Deoxyribose	Carbohydrate Metabolism	PubChem:10786
88	134.0215	15.61	C4H6O5	(R)-Malate	Carbohydrate Metabolism	PubChem:525
89	104.0109	11.37	C3H4O4	2-Hydroxy-3-oxopropanoate	Carbohydrate Metabolism	PubChem:22134427
90	88.0162	8.61	C3H4O3	Pyruvate	Carbohydrate Metabolism	PubChem:107735
91	106.0266	13.78	C3H6O4	D-Glycerate	Carbohydrate Metabolism	PubChem:5460358
92	118.0266	16.91	C4H6O4	Succinate	Carbohydrate Metabolism	PubChem:160419
93	120.0423	12.19	C4H8O4	D-Erythrose	Carbohydrate Metabolism	PubChem:94176
94	130.0266	13.34	C5H6O4	Itaconate	Carbohydrate Metabolism	PubChem:5459996
95	132.0422	16.45	C5H8O4	2-Acetolactate	Carbohydrate Metabolism	PubChem:22

	Mass	RT	Formula	Putative metabolite	Map	PubChem ID
96	134.0215	17.73	C4H6O5	(S)-Malate	Carbohydrate Metabolism	PubChem:525
97	136.0372	14.33	C4H8O5	[FA trihydroxy(4:0)] 2,3,4-trihydroxy-butanoic	Carbohydrate Metabolism	PubChem:439535
98	146.0215	17.23	C5H6O5	2-Oxoglutarate	Carbohydrate Metabolism	PubChem:51
99	150.0527	10.52	C5H10O5	D-Xylulose	Carbohydrate Metabolism	PubChem:5289590
100	162.0528	15.69	C6H10O5	2-Dehydro-3-deoxy-L-rhamnonate	Carbohydrate Metabolism	PubChem:49852300
101	167.9824	18.98	C3H5O6P	Phosphoenolpyruvate	Carbohydrate Metabolism	PubChem:3674425
102	174.0164	19.77	C6H6O6	cis-Aconitate	Carbohydrate Metabolism	PubChem:643757
103	176.0320	16.05	C6H8O6	D-Glucuronolactone	Carbohydrate Metabolism	PubChem:219402
104	178.0477	14.47	C6H10O6	D-Glucono-1,5-lactone	Carbohydrate Metabolism	PubChem:7027
105	180.0634	14.65	C6H12O6	D-Glucose	Carbohydrate Metabolism	PubChem:5793
106	182.0791	10.56	C6H14O6	D-Sorbitol	Carbohydrate Metabolism	PubChem:5780
107	192.0271	19.75	C6H8O7	Citrate	Carbohydrate Metabolism	PubChem:31348
108	196.0583	15.84	C6H12O7	D-Gluconic acid	Carbohydrate Metabolism	PubChem:10690
109	200.0084	16.80	C4H9O7P	D-Erythrose 4-phosphate	Carbohydrate Metabolism	PubChem:122357
110	230.0190	16.80	C5H11O8P	D-Ribose 5-phosphate	Carbohydrate Metabolism	PubChem:439167
111	383.5577	15.18	C21H36N7O16P3S	CoA	Carbohydrate Metabolism	PubChem:87642
112	468.0637	16.50	C14H22N4O8P2S	2-(alpha-Hydroxyethyl)thiamine diphosphate	Carbohydrate Metabolism	PubChem:440568
113	185.9929	18.53	C3H7O7P	3-Phospho-D-glycerate	Carbohydrate Metabolism	PubChem:25245548
114	150.0527	10.52	C5H10O5	D-Ribose	Carbohydrate Metabolism	PubChem:854
115	335.0790	15.28	C11H17N3O7S	S-Formylglutathione	Energy Metabolism	PubChem:25246250
116	427.0295	17.11	C10H15N5O10P2	ADP	Energy Metabolism	PubChem:6022
117	506.9960	18.71	C10H16N5O13P3	ATP	Energy Metabolism	PubChem:5957
118	663.1092	15.99	C21H27N7O14P2	NAD+	Energy Metabolism	PubChem:10897651
119	143.0405	7.01	C6H9NOS	5-(2-Hydroxyethyl)-4-methylthiazole	Metabolism of Cofactors and Vitamins	PubChem:1136
120	115.0269	16.44	C4H5NO3	Maleamate	Metabolism of Cofactors and Vitamins	PubChem:5460391
121	122.0480	7.06	C6H6N2O	Nicotinamide	Metabolism of Cofactors and Vitamins	PubChem:936
122	123.0321	7.79	C6H5NO2	Nicotinate	Metabolism of Cofactors and Vitamins	PubChem:937
123	144.0422	12.41	C6H8O4	2,3-Dimethylmaleate	Metabolism of Cofactors and Vitamins	PubChem:5459894
124	167.0582	7.96	C8H9NO3	Pyridoxal	Metabolism of Cofactors and Vitamins	PubChem:1050
125	168.0897	12.44	C8H12N2O2	Pyridoxamine	Metabolism of Cofactors and Vitamins	PubChem:1052
126	187.1207	11.32	C9H17NO3	8-Amino-7-oxononanoate	Metabolism of Cofactors and Vitamins	PubChem:5460197
127	253.0811	16.22	C9H11N5O4	Neopterin	Metabolism of Cofactors and Vitamins	PubChem:444632

	Mass	RT	Formula	Putative metabolite	Map	PubChem ID
128	254.0901	25.06	C11H14N2O5	N-Ribosylnicotinamide	Metabolism of Cofactors and Vitamins	PubChem:439924
129	264.1046	20.65	C12H16N4OS	Thiamin	Metabolism of Cofactors and Vitamins	PubChem:1130
130	344.0709	16.10	C12H17N4O4PS	Thiamin monophosphate	Metabolism of Cofactors and Vitamins	PubChem:15942892
131	114.0430	7.81	C4H6N2O2	5,6-Dihydrouracil	Nucleotide Metabolism	PubChem:649
132	268.0807	10.54	C10H12N4O5	Inosine	Nucleotide Metabolism	PubChem:6021
133	112.0273	22.16	C4H4N2O2	Orotate(Fragment)	Nucleotide Metabolism	PubChem:967
134	111.0432	14.75	C4H5N3O	Cytosine	Nucleotide Metabolism	PubChem:597
135	97.9674	19.38	H2O4S	Sulfate	Nucleotide Metabolism	PubChem:1117
136	112.0273	8.49	C4H4N2O2	Uracil	Nucleotide Metabolism	PubChem:1174
137	125.0589	7.70	C5H7N3O	5-Methylcytosine	Nucleotide Metabolism	PubChem:65040
138	126.0430	7.27	C5H6N2O2	Thymine	Nucleotide Metabolism	PubChem:1135
139	128.0585	16.29	C5H8N2O2	5,6-Dihydrothymine	Nucleotide Metabolism	PubChem:93556
140	135.0545	9.79	C5H5N5	Adenine	Nucleotide Metabolism	PubChem:190
141	136.0386	10.64	C5H4N4O	Hypoxanthine	Nucleotide Metabolism	PubChem:790
142	151.0493	13.20	C5H5N5O	Guanine	Nucleotide Metabolism	PubChem:764
143	152.0334	12.44	C5H4N4O2	Xanthine	Nucleotide Metabolism	PubChem:1188
144	251.1017	7.94	C10H13N5O3	Deoxyadenosine	Nucleotide Metabolism	PubChem:13730
145	305.0411	14.20	C9H12N3O7P	2',3'-Cyclic CMP	Nucleotide Metabolism	PubChem:53481030
146	322.0567	14.91	C10H15N2O8P	dTMP	Nucleotide Metabolism	PubChem:9700
147	323.0519	17.54	C9H14N3O8P	CMP	Nucleotide Metabolism	PubChem:6131
148	324.0356	16.88	C9H13N2O9P	Pseudouridine 5'-phosphate	Nucleotide Metabolism	PubChem:439424
149	329.0527	10.77	C10H12N5O6P	3',5'-Cyclic AMP	Nucleotide Metabolism	PubChem:6076
150	347.0632	20.81	C10H14N5O7P	dGMP	Nucleotide Metabolism	PubChem:6994968
151	244.0695	12.83	C9H12N2O6	Uridine	Nucleotide Metabolism	PubChem:6029
152	188.0797	9.79	C7H12N2O4	N-Acetylglutamine	Others	PubChem:25561
153	136.0736	9.90	C5H12O4	D-Apiitol	Others	PubChem:151720
154	218.0555	14.13	C13H21N6O9P	(L-Seryl)adenylate	Others	PubChem:25244014
155	255.2562	5.42	C16H33NO	Palmiticamide	Others	PubChem:69421
156	143.0582	8.16	C6H9NO3	Vinylacetylglycine	Others	PubChem:53477718
157	118.0266	6.46	C4H6O4	Methyl oxalate	Others	PubChem:11120
158	246.0851	9.72	C9H14N2O6	5-6-Dihydrouridine	Others	PubChem:94312
159	129.1518	7.82	C8H19N	Octylamine	Others	PubChem:8143

	Mass	RT	Formula	Putative metabolite	Map	PubChem ID
160	227.2249	5.47	C14H29NO	Myristic amide	Others	PubChem:69492
161	257.2355	6.85	C15H31NO2	Decanoylcholine	Others	PubChem:9974373
162	85.0891	11.41	C5H11N	Piperidine	Others	PubChem:849
163	133.0197	14.27	C4H7NO2S	L-Thiazolidine-4-carboxylate	Others	PubChem:9934
164	189.0790	7.63	C11H11NO2	3-Indolepropionicacid	Others	PubChem:3744
165	243.1834	5.67	C13H25NO3	N-Undecanoylglycine	Others	PubChem:454092
166	257.1011	9.21	C10H15N3O5	5-Methylcytidine	Others	PubChem:92918
167	215.1521	5.80	C11H21NO3	N-Nonanoylglycine	Others	PubChem:10176752
168	100.0160	7.02	C4H4O3	2-Oxobut-3-enanoate	Others	PubChem:9543327
169	116.0838	4.58	C6H12O2	4-Hydroxyhexan-3-one	Others	PubChem:95609
170	127.0633	26.05	C6H9NO2	Alpha-(Methylenecyclopropyl)glycine	Others	PubChem:25203707
171	132.0900	12.91	C5H12N2O2	N4-acetyl-N4-hydroxy-1-aminopropane	Others	PubChem:25201397
172	133.0739	6.98	C5H11NO3	N-hydroxyvaline	Others	PubChem:95562
173	133.0739	14.74	C5H11NO3	1-deoxyxylonojirimycin	Others	PubChem:25203271
174	143.0945	11.36	C7H13NO2	Stachydrine	Others	PubChem:554
175	153.9935	18.08	C3H6O5S	3-sulfopropanoate	Others	PubChem:22461918
176	156.0189	13.29	C3H9O5P	Propane-1,2-diol 1-phosphate	Others	PubChem:18179361
177	160.1211	17.34	C7H16N2O2	L-Carnitinamide	Others	PubChem:4633873
178	163.0495	13.66	C6H5N5O	Pterin	Others	PubChem:73000
179	163.0844	12.07	C6H13NO4	1-deoxynojirimycin	Others	PubChem:29435
180	165.0652	13.66	C6H7N5O	3-Methylguanine	Others	PubChem:76292
181	169.0849	7.42	C7H11N3O2	histidine methyl ester	Others	PubChem:92893
182	169.9885	18.94	C3H6O6S	3-Sulfolactate	Others	PubChem:25203561
183	181.0601	13.04	C6H7N5O2	8-Hydroxy-7-methylguanine	Others	PubChem:308075
184	181.0964	10.66	C7H11N5O	6-methyltetrahydropterin	Others	PubChem:3124
185	187.1208	4.89	C9H17NO3	N-Heptanoylglycine	Others	PubChem:10932172
186	187.1685	30.00	C9H21N3O	N1-Acetylspermidine	Others	PubChem:496
187	189.1113	18.73	C7H15N3O3	L-Homocitrulline	Others	PubChem:65072
188	191.0617	6.96	C7H13NO3S	N-Acetylmethionine	Others	PubChem:448580
189	197.0801	11.61	C8H11N3O3	N-Acetyl-L-histidine	Others	PubChem:25201295
190	201.1363	4.68	C10H19NO3	Capryloylglycine	Others	PubChem:84290
191	216.1224	16.26	C8H16N4O3	N-acetyl-(L)-arginine	Others	PubChem:67427

	Mass	RT	Formula	Putative metabolite	Map	PubChem ID
192	229.1678	4.47	C12H23NO3	N-Decanoylglycine	Others	PubChem:1712391
193	257.1991	4.22	C14H27NO3	N-Lauroylglycine	Others	PubChem:346152
194	271.2147	4.11	C15H29NO3	Tridecanoylglycine	Others	PubChem:45357453
195	278.1517	3.70	C16H22O4	2-Ethylhexyl phthalate	Others	PubChem:20393
196	422.2346	3.49	C20H38O7S	1,4-Bis(2-ethylhexyl) sulfosuccinate	Others	PubChem:11339
197	424.0372	17.11	C14H18NO10S2	Sinalbin	Others	PubChem:10621
198	541.0611	15.97	C15H21N5O13P2	Cyclic ADP-ribose	Others	PubChem:123847
