# Supplementary material for: LC–MS-based absolute metabolite quantification: application to metabolic flux measurement in trypanosomes
Source: Metabolomics. 2015 Jul 9;11(6):1721–32. doi: 10.1007/s11306-015-0827-2 (PMC4605981; doi:10.1007/s11306-015-0827-2)
Supplement: Supplementary file 4 — Supplementary material 4 (PDF 91 kb). Supplementary table S4 – PDF of concentrations of extracellular metabolites from spent CMM media where T. brucei blood stream forms were grown at different time points (0, 7, 24, 31, 48, 51 and 57 h) [file 11306_2015_827_MOESM4_ESM.pdf]

SUPPLEMENTARY INFORMATION

LC-MS-based absolute metabolite quantification: Application to metabolic flux measurement in trypanosomes

Dong-Hyun Kim[1,2], Fiona Achcar[1], Rainer Breitling[3], Karl E. Burgess[4], Michael P. Barrett[1,4*]

[1]Wellcome Trust Centre for Molecular Parasitology, Institute of Infection, Immunity and Inflammation, College of Medical Veterinary and Life Sciences, University of Glasgow, G12 8TA, UK

[2]Current: Centre for Analytical Bioscience, School of Pharmacy, University of Nottingham, University Park, Nottingham, NG7 2RD, UK

[3]Manchester Centre of Synthetic Biology for Fine and Speciality Chemicals, Manchester Institute of Biotechnology, Faculty of Life Sciences, University of Manchester, Manchester, M1 7DN, UK.

[4]Glasgow Polyomics, Wolfson Wohl Cancer Research Centre, College of Medical Veterinary & Life Sciences, University of Glasgow, G61 1QH, UK

*Address for Correspondence:

M.P. Barrett, Wellcome Trust Centre for Molecular Parasitology, Institute of Infection, Immunity and Inflammation, College of Medical Veterinary and Life Sciences, University of Glasgow, G12 8TA, UK
E-mail: Michael.Barrett@glasgow.ac.uk
Tel: +44 (0) 141 330 6904. Fax: +44 (0) 141 330 4077

Name	Extracellular concentration (µM ± SEM)						
	0h	7h	24h	31h	48h	51h	57h
Inosine	1.8 ± 0.1	1.8 ± 0.1	1.9 ± 0.1	2.1 ± 0.1	0.9 ± 0.1	-	-
L-Arginine	142.8 ± 6.1	142 ± 3.5	148.1 ± 3.1	168.0 ± 6.8	157.5 ± 3.2	157.9 ± 0.9	156.1 ± 5.8
L-Asparagine	56.8 ± 2.8	56.9 ± 1.9	71.0 ± 1.9	84.0 ± 3.1	87.7 ± 2.2	87.5 ± 0.7	85.3 ± 2.8
L-Aspartate	47.5 ± 2.4	48.7 ± 2.2	60.2 ± 1.7	71.5 ± 3.0	77.7 ± 2.4	78.9 ± 0.6	80.5 ± 3.1
L-Glutamine	790.9 ± 28.8	797.3 ± 12.0	733.8 ± 10.4	785.4 ± 33.6	660.8 ± 14.8	624.1 ± 9.9	562.0 ± 15.0
L-Histidine	22.9 ± 1.1	23.4 ± 0.8	27.6 ± 0.8	31.7 ± 1.3	32.8 ± 1.1	31.9 ± 0.3	32.0 ± 0.7
L-Lysine	192.1 ± 7.2	192.8 ± 4.5	203.9 ± 3.3	227.6 ± 9.0	217.5 ± 4.4	214.4 ± 1.2	210.9 ± 6.6
L-Methionine	22.5 ± 1.0	22.9 ± 0.7	25.9 ± 0.5	30.2 ± 1.2	26.5 ± 0.4	24.6 ± 0.7	17.9 ± 0.6
L-Proline	59.3 ± 2.4	59.3 ± 1.8	72.7 ± 1.4	86.6 ± 4.4	85.2 ± 1.8	85.0 ± 0.7	83.8 ± 3.5
L-Threonine	62.6 ± 2.8	63.6 ± 2.2	75.1 ± 1.7	86.9 ± 4.1	80.7 ± 1.4	78.1 ± 1.2	67.6 ± 1.8
L-Tryptophan	10.2 ± 1.1	8.8 ± 0.3	10.1 ± 0.3	12.1 ± 0.8	7.6 ± 0.3	5.9 ± 0.5	0.8 ± 0.1
L-Valine	87.2 ± 3.6	87.6 ± 3.5	108.2 ± 3.9	126.7 ± 3.8	114.9 ± 2.9	108.5 ± 1.4	88.0 ± 2.1
Pyruvate	61.7 ± 2.0	92.5 ± 7.2	240.2 ± 30.3	652.4 ± 56.8	5467.9 ± 472.0	7740.3 ± 493.5	15683.9 ± 1437.6
L-Glutamate	79.3 ± 4.2	80.3 ± 3.0	93.4 ± 2.5	113.7 ± 5.3	131.2 ± 5.2	137.8 ± 1.9	151.8 ± 7.4
cis-Aconitate	2.0 ± 0.1	2.0 ± 0.0	2.0 ± 0.0	2.1 ± 0.1	2.0 ± 0.0	2.0 ± 0.1	2.1 ± 0.1
Xanthine	185.7 ± 6.9	188.4 ± 4.0	196.7 ± 3.2	225.8 ± 9.0	240.9 ± 6.3	251.5 ± 2.7	269.2 ± 12.5
L-Homoserine	23.9 ± 1.2	24.3 ± 0.9	29.3 ± 0.8	34.6 ± 1.9	31.8 ± 0.6	30.6 ± 0.5	26.1 ± 0.8
L-Tyrosine	89.1 ± 4.0	89.2 ± 3.3	111.1 ± 2.2	131.1 ± 5.7	120.9 ± 1.8	113.8 ± 1.7	80.8 ± 2.5
L-Ornithine	15.9 ± 0.7	16.8 ± 1.1	16.6 ± 0.7	18.1 ± 1.0	17.1 ± 0.7	16.7 ± 0.2	16.0 ± 0.6
Pantothenate	1.6 ± 0.1	1.6 ± 0.0	1.5 ± 0.0	1.7 ± 0.1	1.6 ± 0.1	1.7 ± 0.0	1.7 ± 0.1
L-Alanine	356.3 ± 52.9	331.1 ± 59.7	333.6 ± 22.6	392.2 ± 37.9	540.9 ± 22.3	606.7 ± 40.1	761.4 ± 84.5
2-Oxoglutarate	0.8 ± 0.1	0.9 ± 0.1	1.2 ± 0.1	1.9 ± 0.1	13.0 ± 1.3	18.8 ± 1.2	39.9 ± 3.5
Succinate	2.8 ± 0.1	2.9 ± 0.1	2.8 ± 0.0	3.3 ± 0.1	5.1 ± 0.3	6.3 ± 0.3	11.5 ± 1.1
Citrate	212.8 ± 8.4	214.5 ± 4.6	207.1 ± 5.2	231.2 ± 10.9	216.5 ± 5.4	216.3 ± 5.1	223.4 ± 8.8
Hypoxanthine	11.9 ± 0.7	11.7 ± 0.5	12.5 ± 0.4	14.3 ± 0.5	6.1 ± 0.4	3.4 ± 0.5	-
O-Acetyl-L-serine	1.0 ± 0.1	1.1 ± 0.1	1.1 ± 0.1	1.2 ± 0.1	1.2 ± 0.1	1.2 ± 0.0	1.3 ± 0.1
Glycerate	1.8 ± 0.1	1.9 ± 0.0	1.9 ± 0.0	1.9 ± 0.0	2.5 ± 0.0	2.7 ± 0.1	3.5 ± 0.2
L-Isoleucine	132.3 ± 6.0	132.2 ± 4.8	168.9 ± 4.5	189.6 ± 6.5	128.9 ± 2.8	107.0 ± 3.9	61.4 ± 2.0
Glycine	143.4 ± 8.2	146.9 ± 3.8	161.7 ± 3.0	183.3 ± 6.1	186.8 ± 3.6	186.9 ± 1.5	195.5 ± 7.2
L-Phenylalanine	55.4 ± 13.4	43.4 ± 4.3	78.8 ± 21.8	56.1 ± 3.3	68.3 ± 33.4	42.6 ± 10.7	2.9 ± 0.7

Name	Extracellular concentration (µM ± SEM)						
	0h	7h	24h	31h	48h	51h	57h
L-Citrulline	4.0 ± 0.2	4.1 ± 0.1	3.9 ± 0.1	4.3 ± 0.2	4.0 ± 0.1	4.1 ± 0.0	4.2 ± 0.2
L-Serine	118.5 ± 4.0	121.7 ± 4.1	133.3 ± 2.9	145.5 ± 4.9	146.5 ± 2.3	146.0 ± 2.9	148.0 ± 5.1
