# Supplementary material for: LC–MS-based absolute metabolite quantification: application to metabolic flux measurement in trypanosomes
Source: Metabolomics. 2015 Jul 9;11(6):1721–32. doi: 10.1007/s11306-015-0827-2 (PMC4605981; doi:10.1007/s11306-015-0827-2)
Supplement: Supplementary file 5 — Supplementary material 5 (PDF 155 kb). Supplementary figure S6 – PDF of concentrations of metabolites from the time courses identified in CMM medium that could not be quantified. Each colour corresponds to a biological replicate of the time course [file 11306_2015_827_MOESM5_ESM.pdf]

SUPPLEMENTARY INFORMATION

LC-MS-based absolute metabolite quantification: Application to metabolic flux measurement in trypanosomes

Dong-Hyun Kim[1,2], Fiona Achcar[1], Rainer Breitling[3], Karl E. Burgess[4], Michael P. Barrett[1,4*]

[1]Wellcome Trust Centre for Molecular Parasitology, Institute of Infection, Immunity and Inflammation, College of Medical Veterinary and Life Sciences, University of Glasgow, G12 8TA, UK

[2]Current: Centre for Analytical Bioscience, School of Pharmacy, University of Nottingham, University Park, Nottingham, NG7 2RD, UK

[3]Manchester Centre of Synthetic Biology for Fine and Speciality Chemicals, Manchester Institute of Biotechnology, Faculty of Life Sciences, University of Manchester, Manchester, M1 7DN, UK.

[4]Glasgow Polyomics, Wolfson Wohl Cancer Research Centre, College of Medical Veterinary & Life Sciences, University of Glasgow, G61 1QH, UK

*Address for Correspondence:

M.P. Barrett, Wellcome Trust Centre for Molecular Parasitology, Institute of Infection, Immunity and Inflammation, College of Medical Veterinary and Life Sciences, University of Glasgow, G12 8TA, UK
E-mail: Michael.Barrett@glasgow.ac.uk
Tel: +44 (0) 141 330 6904. Fax: +44 (0) 141 330 4077

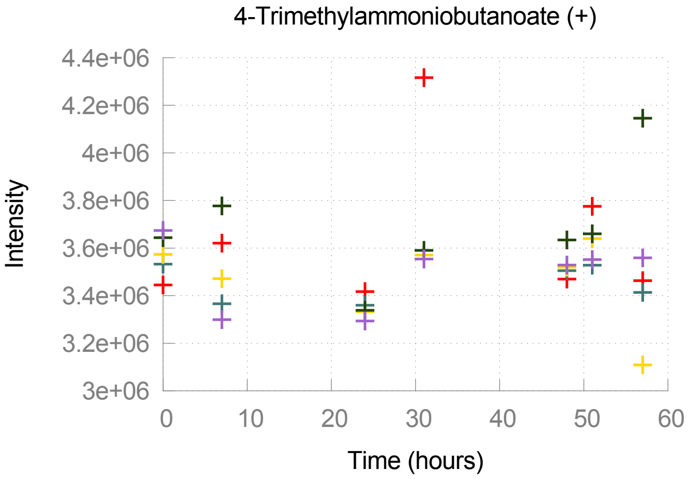
4-Trimethylammoniobutanoate (+)
Intensity
Time (hours)

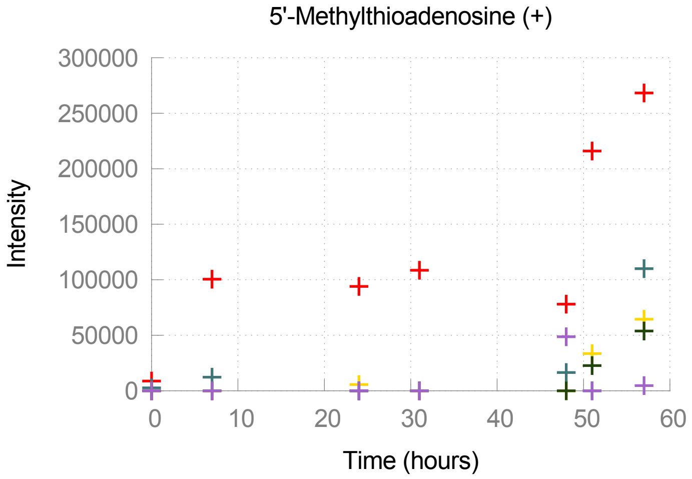
5'-Methylthioadenosine (+)
Intensity
Time (hours)

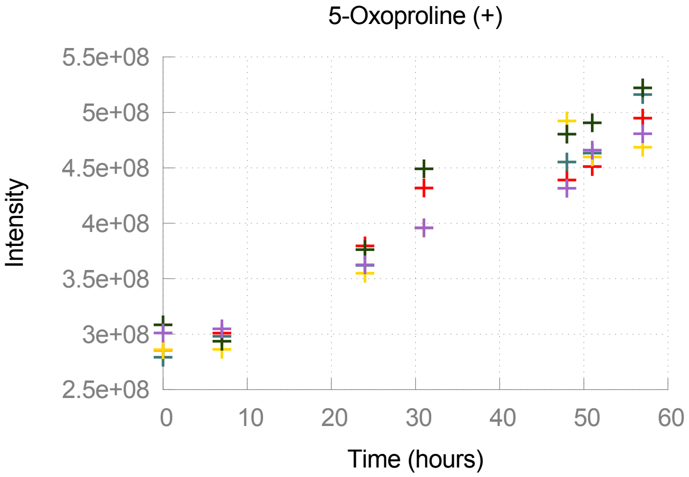
5-Oxoproline (+)
Intensity
Time (hours)

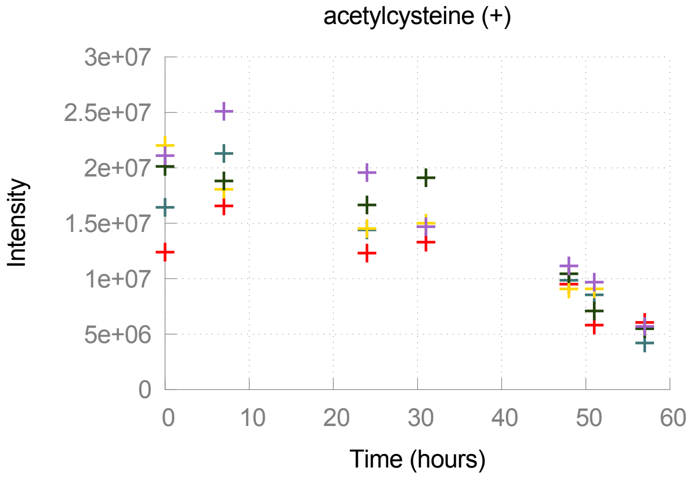
acetylcysteine (+)
Intensity
Time (hours)

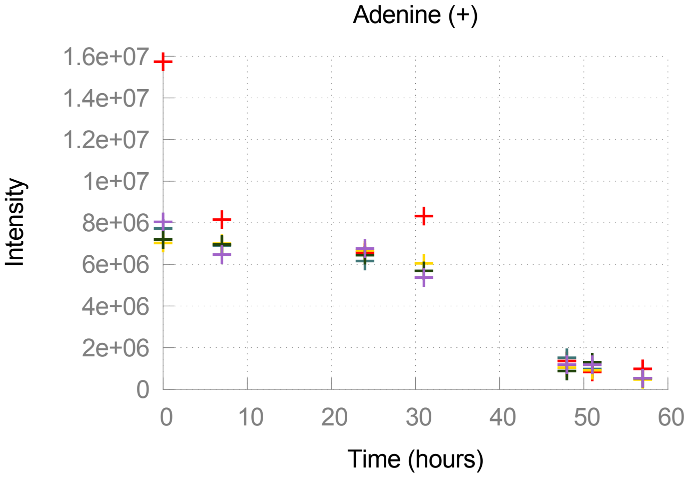
Adenine (+)
Intensity
Time (hours)

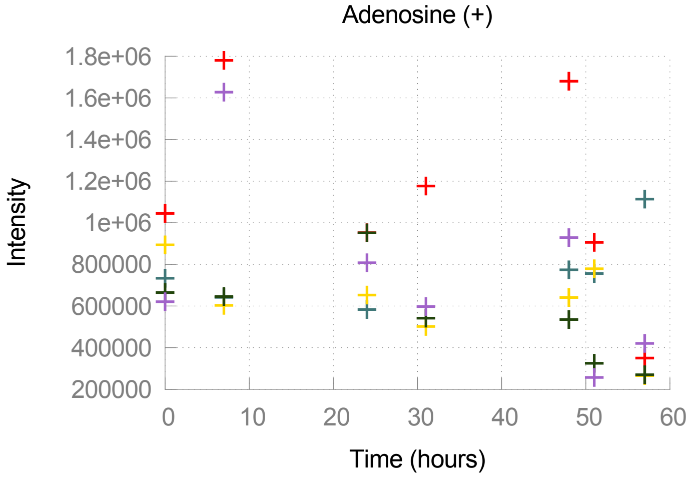
Adenosine (+)
Intensity
Time (hours)

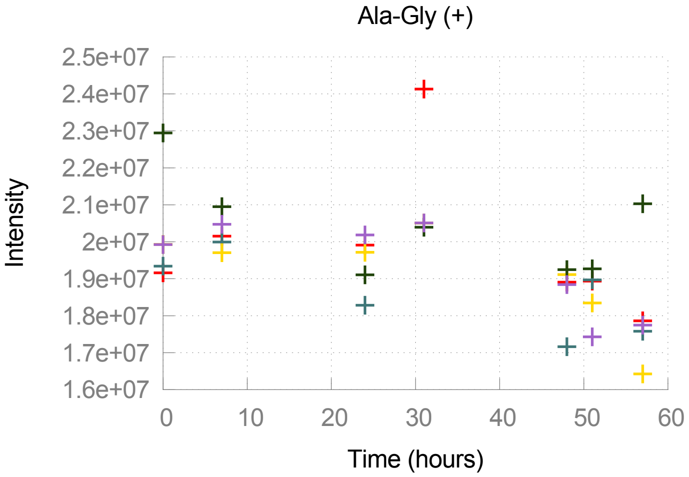
Ala-Gly (+)
Intensity
Time (hours)

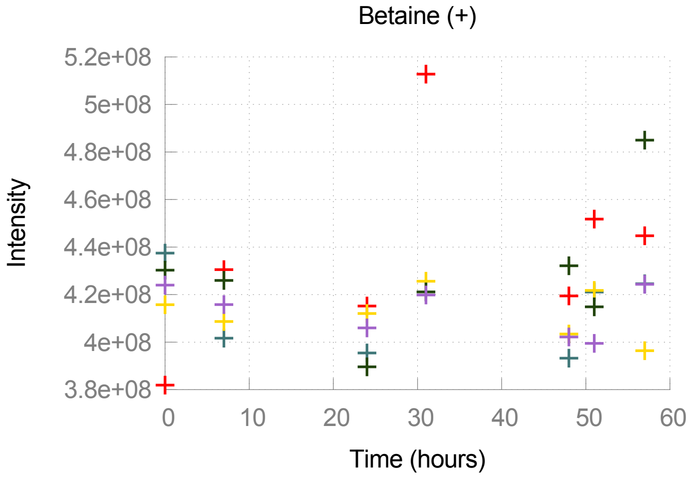
Betaine (+)
Intensity
Time (hours)

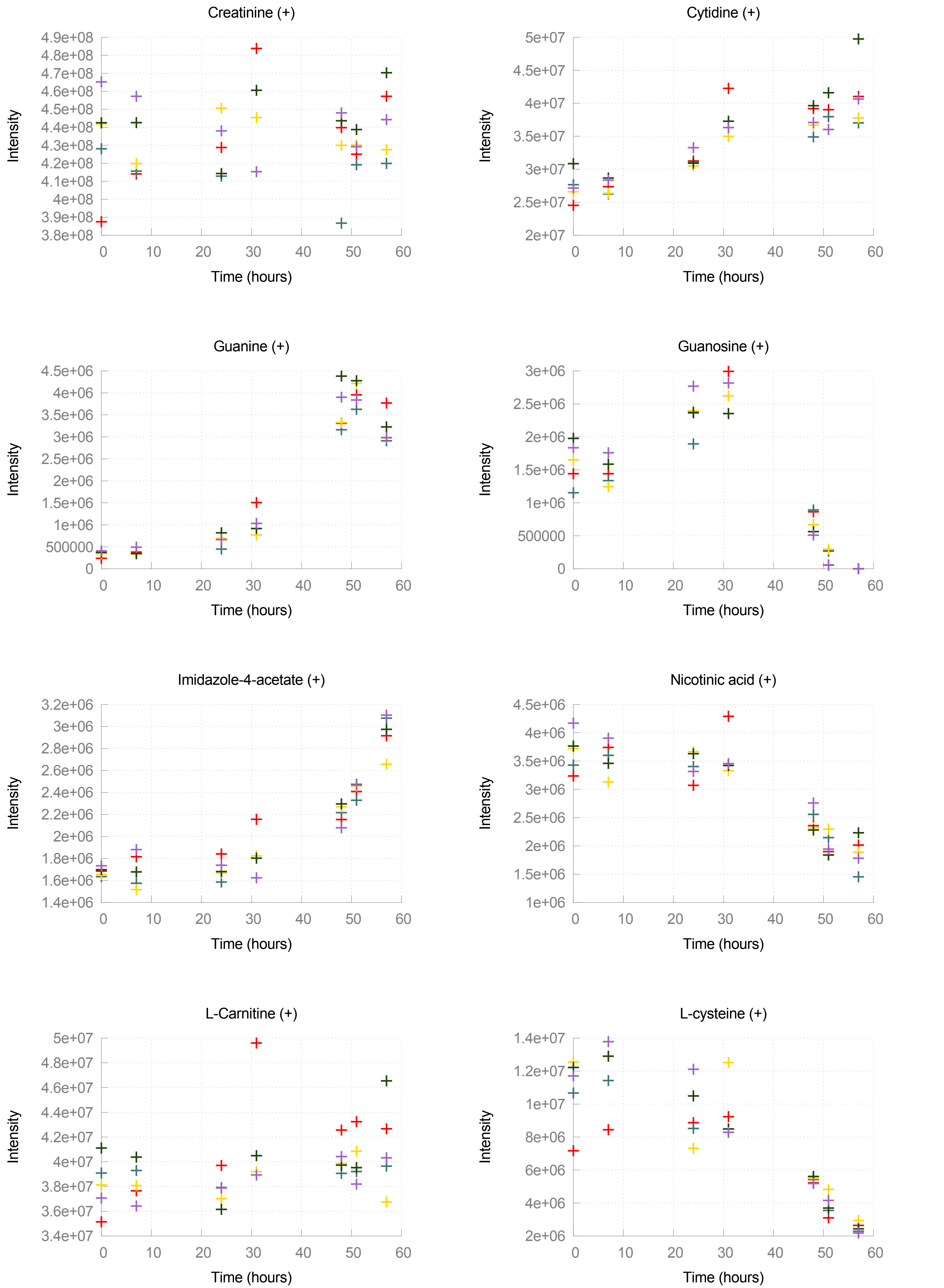
Creatinine (+)
4.9e+08
4.8e+08
4.7e+08
4.6e+08
4.5e+08
4.4e+08
4.3e+08
4.2e+08
4.1e+08
4e+08
3.9e+08
3.8e+08
Intensity
0 10 20 30 40 50 60
Time (hours)
Cytidine (+)
5e+07
4.5e+07
4e+07
3.5e+07
3e+07
2.5e+07
2e+07
Intensity
0 10 20 30 40 50 60
Time (hours)
Guanine (+)
4.5e+06
4e+06
3.5e+06
3e+06
2.5e+06
2e+06
1.5e+06
1e+06
500000
0
Intensity
0 10 20 30 40 50 60
Time (hours)
Guanosine (+)
3e+06
2.5e+06
2e+06
1.5e+06
1e+06
500000
0
Intensity
0 10 20 30 40 50 60
Time (hours)
Imidazole-4-acetate (+)
3.2e+06
3e+06
2.8e+06
2.6e+06
2.4e+06
2.2e+06
2e+06
1.8e+06
1.6e+06
1.4e+06
Intensity
0 10 20 30 40 50 60
Time (hours)
Nicotinic acid (+)
4.5e+06
4e+06
3.5e+06
3e+06
2.5e+06
2e+06
1.5e+06
1e+06
Intensity
0 10 20 30 40 50 60
Time (hours)
L-Carnitine (+)
5e+07
4.8e+07
4.6e+07
4.4e+07
4.2e+07
4e+07
3.8e+07
3.6e+07
3.4e+07
Intensity
0 10 20 30 40 50 60
Time (hours)
L-cysteine (+)
1.4e+07
1.2e+07
1e+07
8e+06
6e+06
4e+06
2e+06
Intensity
0 10 20 30 40 50 60
Time (hours)

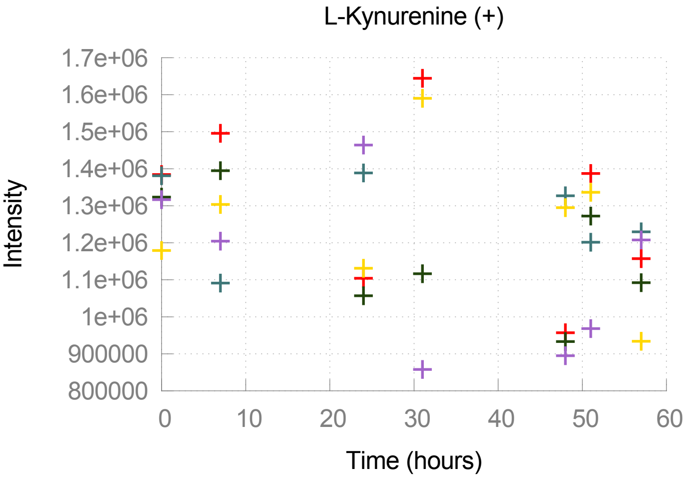
L-Kynurenine (+)
Intensity
1.7e+06
1.6e+06
1.5e+06
1.4e+06
1.3e+06
1.2e+06
1.1e+06
1e+06
900000
800000
0 10 20 30 40 50 60
Time (hours)

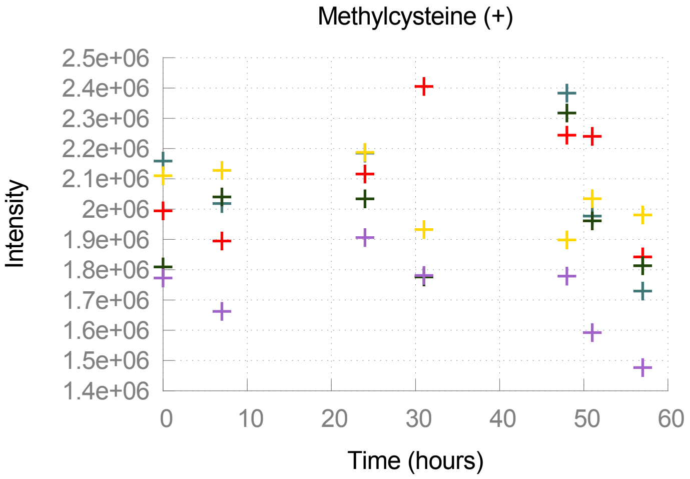
Methylcysteine (+)
Intensity
2.5e+06
2.4e+06
2.3e+06
2.2e+06
2.1e+06
2e+06
1.9e+06
1.8e+06
1.7e+06
1.6e+06
1.5e+06
1.4e+06
0 10 20 30 40 50 60
Time (hours)

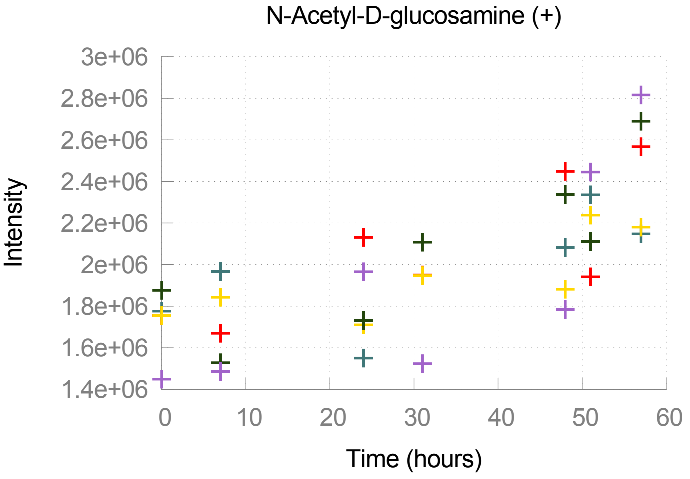
N-Acetyl-D-glucosamine (+)
Intensity
3e+06
2.8e+06
2.6e+06
2.4e+06
2.2e+06
2e+06
1.8e+06
1.6e+06
1.4e+06
0 10 20 30 40 50 60
Time (hours)

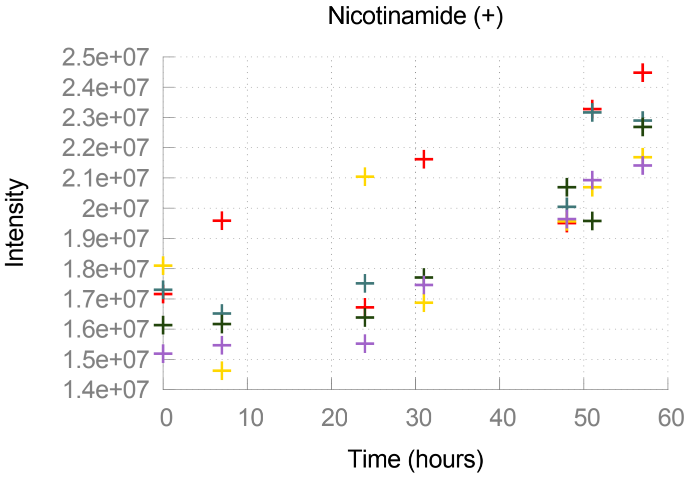
Nicotinamide (+)
Intensity
2.5e+07
2.4e+07
2.3e+07
2.2e+07
2.1e+07
2e+07
1.9e+07
1.8e+07
1.7e+07
1.6e+07
1.5e+07
1.4e+07
0 10 20 30 40 50 60
Time (hours)

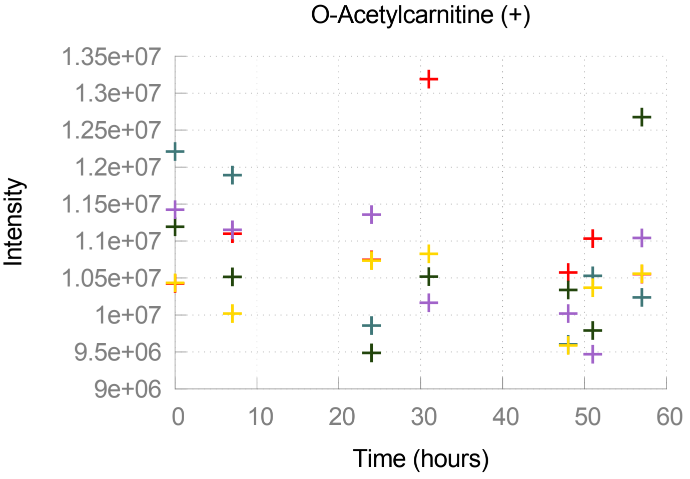
O-Acetylcarnitine (+)
Intensity
1.35e+07
1.3e+07
1.25e+07
1.2e+07
1.15e+07
1.1e+07
1.05e+07
1e+07
9.5e+06
9e+06
0 10 20 30 40 50 60
Time (hours)

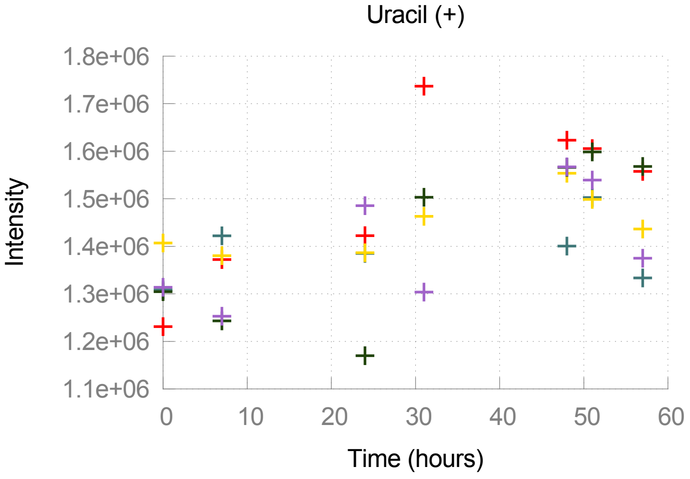
Uracil (+)
Intensity
1.8e+06
1.7e+06
1.6e+06
1.5e+06
1.4e+06
1.3e+06
1.2e+06
1.1e+06
0 10 20 30 40 50 60
Time (hours)

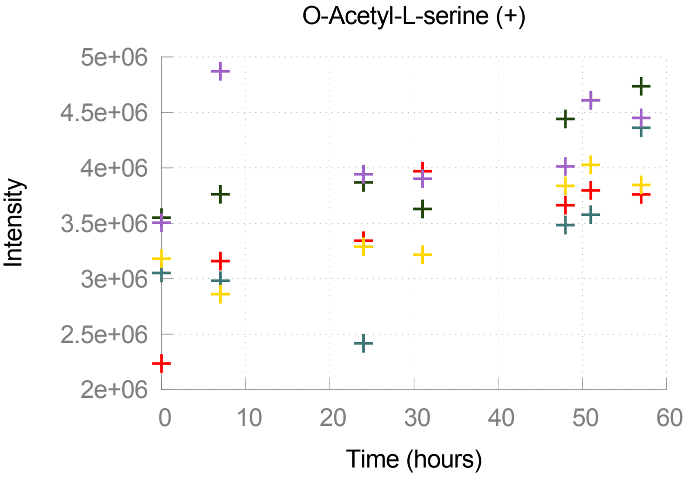
O-Acetyl-L-serine (+)
Intensity
5e+06
4.5e+06
4e+06
3.5e+06
3e+06
2.5e+06
2e+06
0 10 20 30 40 50 60
Time (hours)

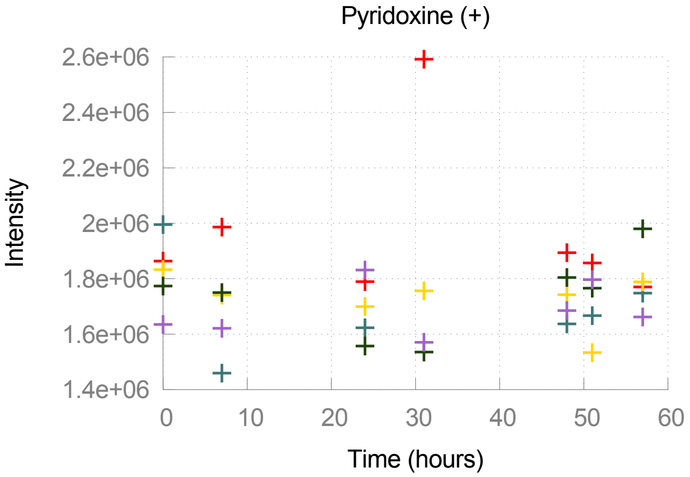
Pyridoxine (+)
Intensity
2.6e+06
2.4e+06
2.2e+06
2e+06
1.8e+06
1.6e+06
1.4e+06
0 10 20 30 40 50 60
Time (hours)

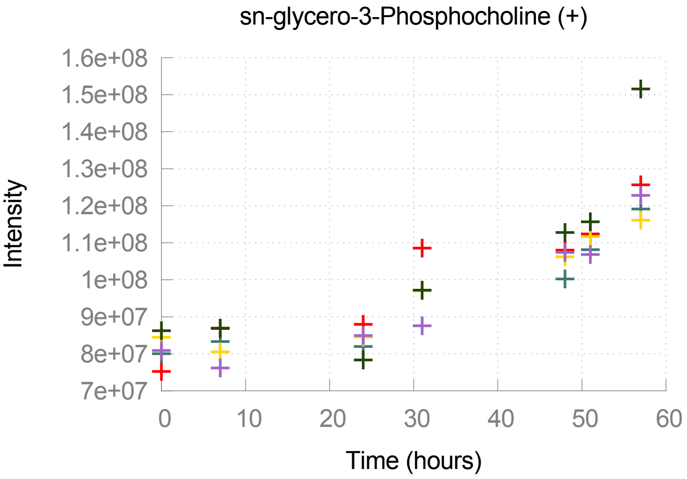
sn-glycero-3-Phosphocholine (+)
Intensity
1.6e+08
1.5e+08
1.4e+08
1.3e+08
1.2e+08
1.1e+08
1e+08
9e+07
8e+07
7e+07
0 10 20 30 40 50 60
Time (hours)

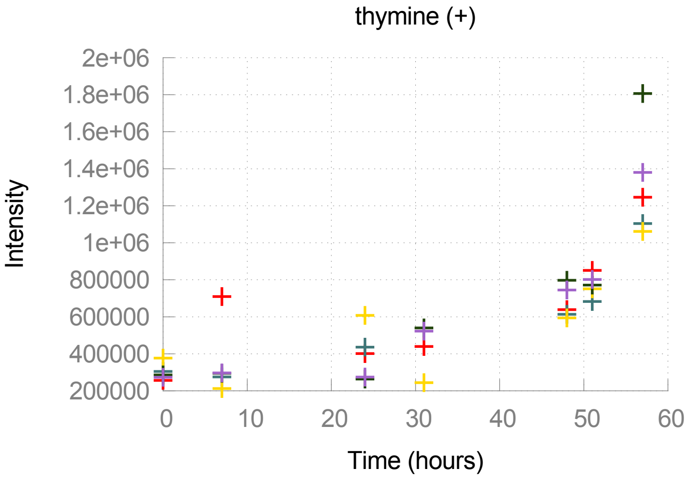
thymine (+)
Intensity
2e+06
1.8e+06
1.6e+06
1.4e+06
1.2e+06
1e+06
800000
600000
400000
200000
0 10 20 30 40 50 60
Time (hours)

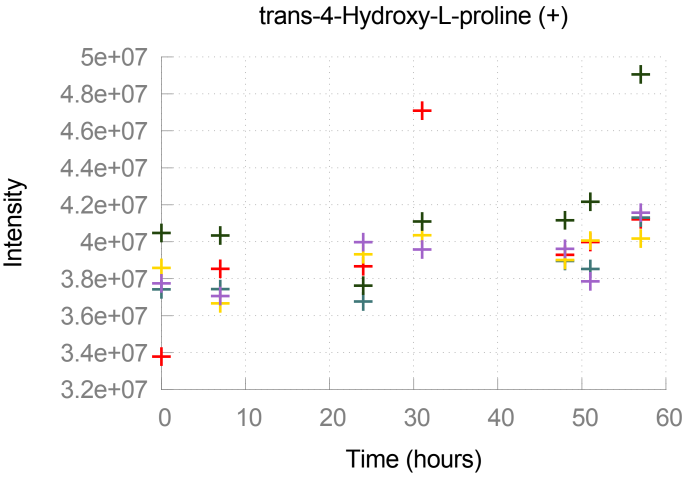
trans-4-Hydroxy-L-proline (+)
Intensity
5e+07
4.8e+07
4.6e+07
4.4e+07
4.2e+07
4e+07
3.8e+07
3.6e+07
3.4e+07
3.2e+07
0 10 20 30 40 50 60
Time (hours)

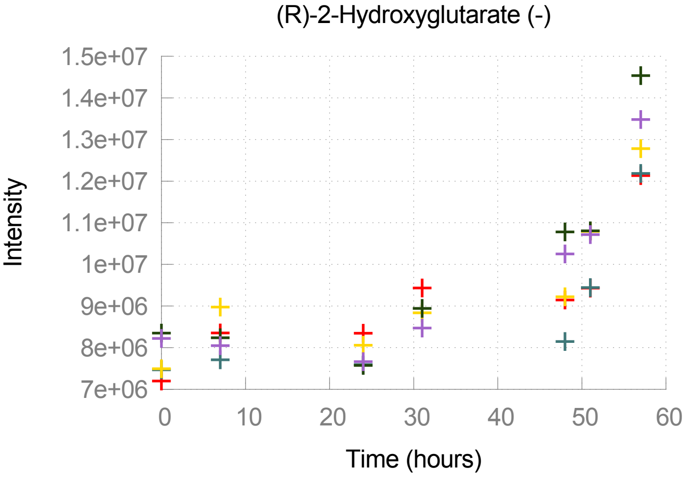
(R)-2-Hydroxyglutarate (-)
Intensity
1.5e+07
1.4e+07
1.3e+07
1.2e+07
1.1e+07
1e+07
9e+06
8e+06
7e+06
0 10 20 30 40 50 60
Time (hours)

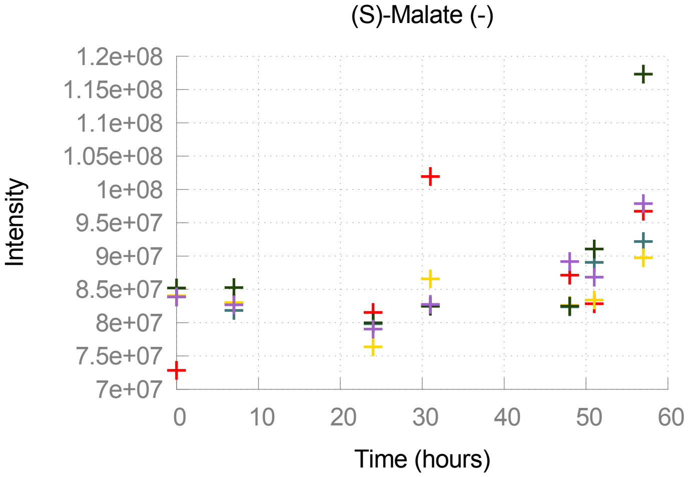
(S)-Malate (-)
Intensity
1.2e+08
1.15e+08
1.1e+08
1.05e+08
1e+08
9.5e+07
9e+07
8.5e+07
8e+07
7.5e+07
7e+07
0 10 20 30 40 50 60
Time (hours)

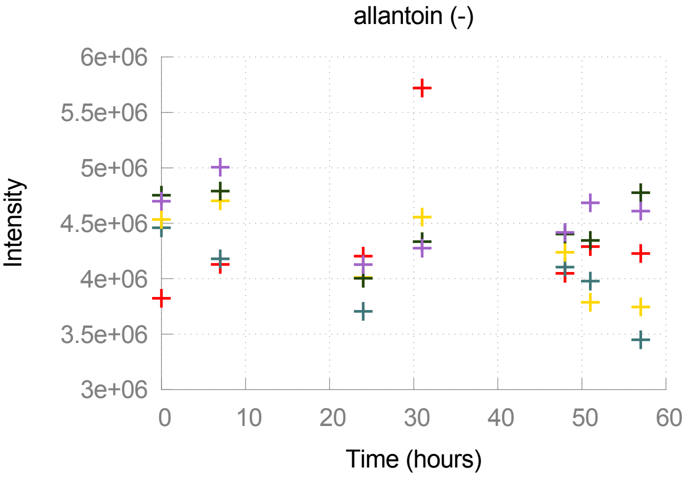
allantoin (-)
Intensity
6e+06
5.5e+06
5e+06
4.5e+06
4e+06
3.5e+06
3e+06
0 10 20 30 40 50 60
Time (hours)

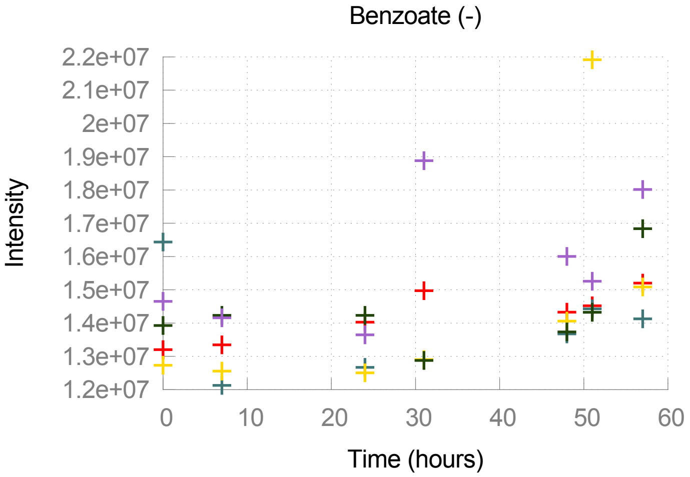
Benzoate (-)
Intensity
2.2e+07
2.1e+07
2e+07
1.9e+07
1.8e+07
1.7e+07
1.6e+07
1.5e+07
1.4e+07
1.3e+07
1.2e+07
0 10 20 30 40 50 60
Time (hours)

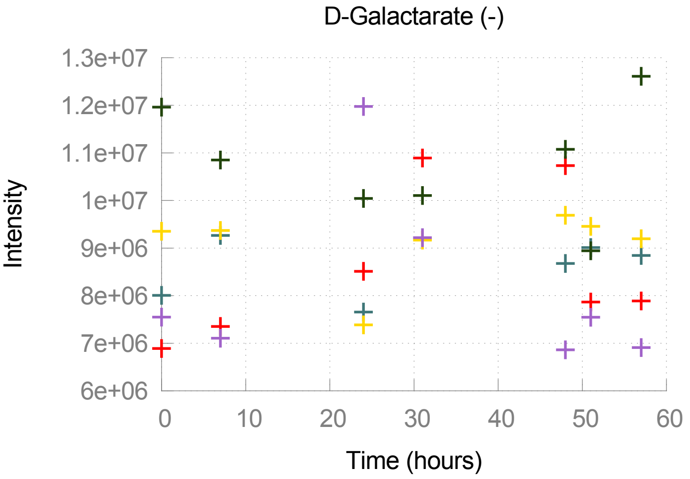
D-Galactarate (-)
Intensity
1.3e+07
1.2e+07
1.1e+07
1e+07
9e+06
8e+06
7e+06
6e+06
0
10
20
30
40
50
60
Time (hours)

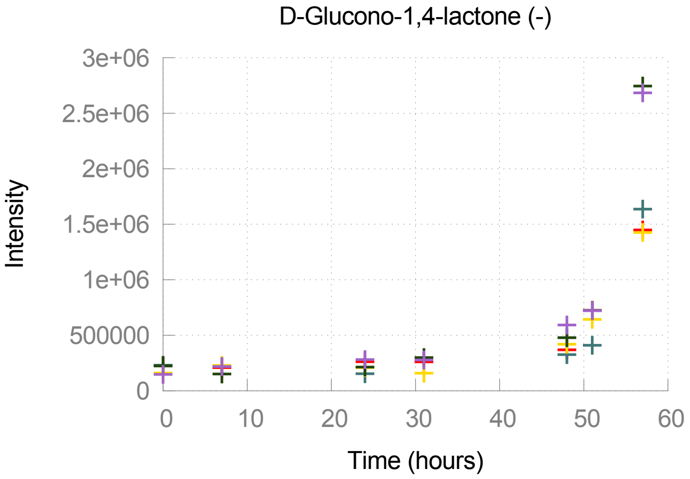
D-Glucono-1,4-lactone (-)
Intensity
3e+06
2.5e+06
2e+06
1.5e+06
1e+06
500000
0
0
10
20
30
40
50
60
Time (hours)

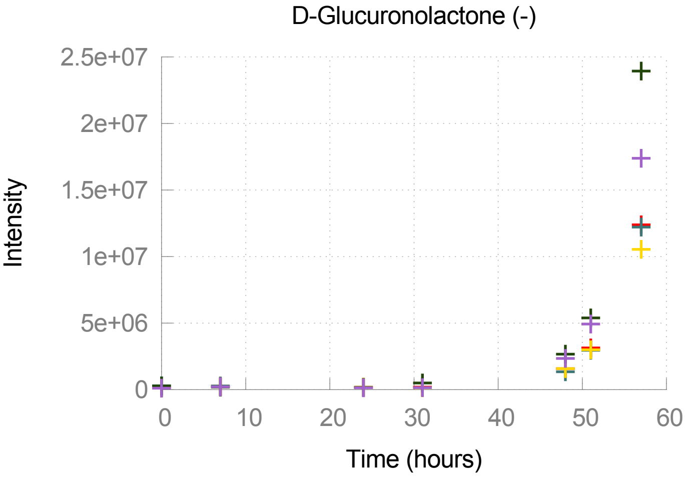
D-Glucuronolactone (-)
Intensity
2.5e+07
2e+07
1.5e+07
1e+07
5e+06
0
0
10
20
30
40
50
60
Time (hours)

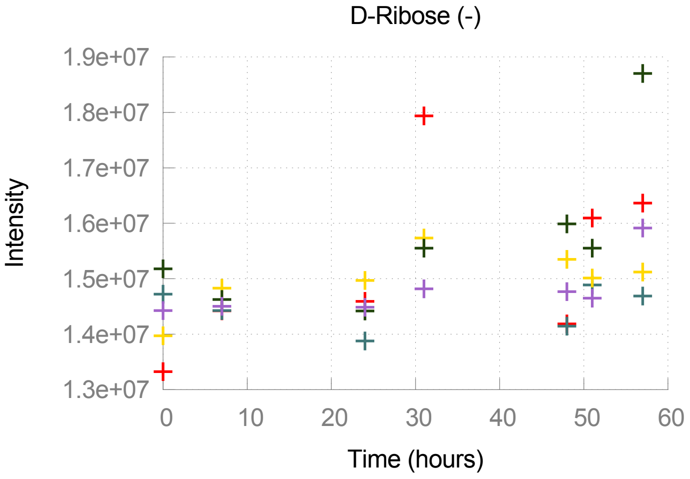
D-Ribose (-)
Intensity
1.9e+07
1.8e+07
1.7e+07
1.6e+07
1.5e+07
1.4e+07
1.3e+07
0
10
20
30
40
50
60
Time (hours)

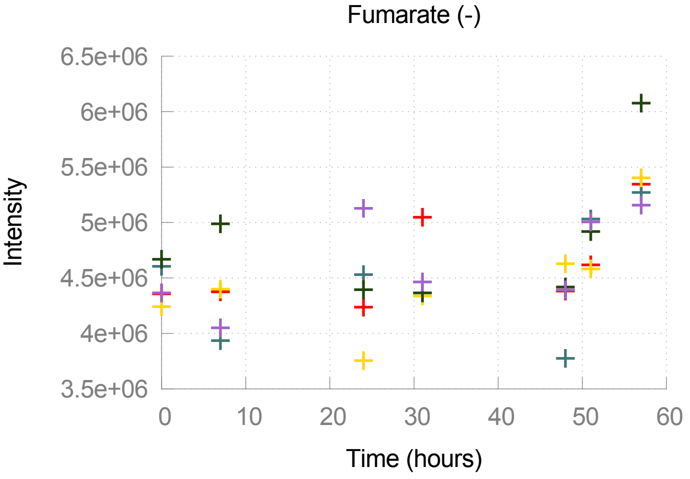
Fumarate (-)
Intensity
6.5e+06
6e+06
5.5e+06
5e+06
4.5e+06
4e+06
3.5e+06
0
10
20
30
40
50
60
Time (hours)

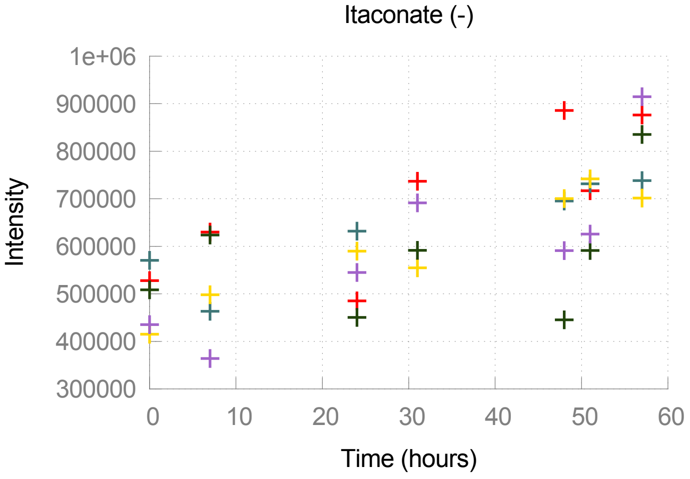
Itaconate (-)
Intensity
1e+06
900000
800000
700000
600000
500000
400000
300000
0
10
20
30
40
50
60
Time (hours)

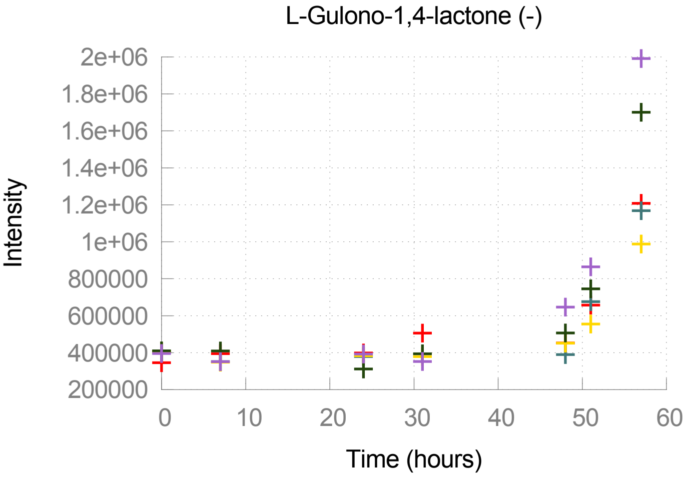
L-Gulono-1,4-lactone (-)
Intensity
2e+06
1.8e+06
1.6e+06
1.4e+06
1.2e+06
1e+06
800000
600000
400000
200000
0
10
20
30
40
50
60
Time (hours)

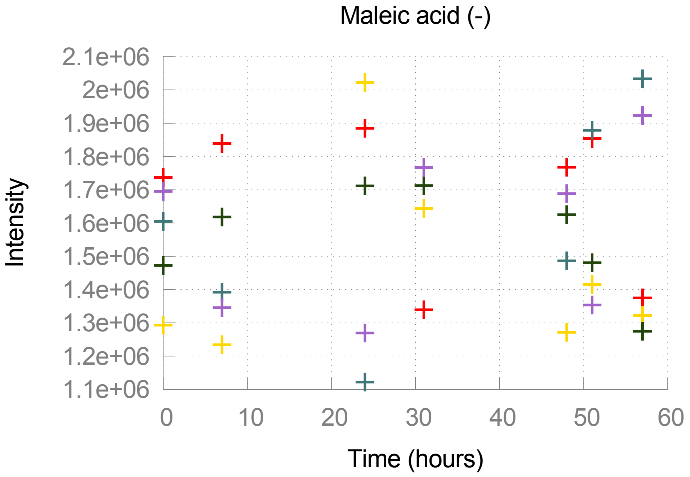
Maleic acid (-)
Intensity
2.1e+06
2e+06
1.9e+06
1.8e+06
1.7e+06
1.6e+06
1.5e+06
1.4e+06
1.3e+06
1.2e+06
1.1e+06
0
10
20
30
40
50
60
Time (hours)

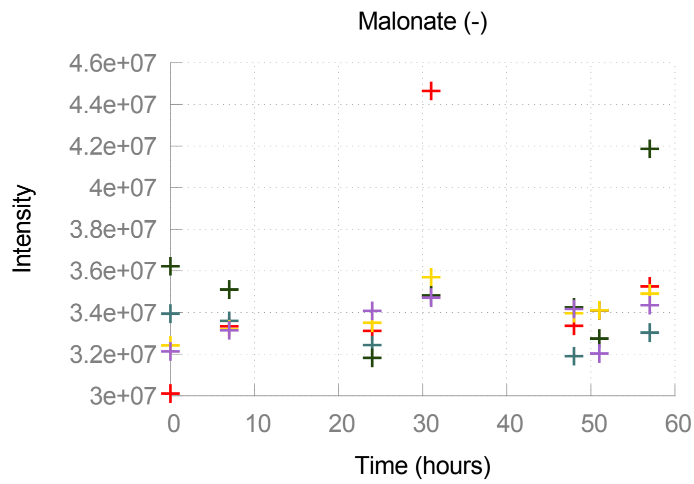
Malonate (-)
Intensity
4.6e+07
4.4e+07
4.2e+07
4e+07
3.8e+07
3.6e+07
3.4e+07
3.2e+07
3e+07
0
10
20
30
40
50
60
Time (hours)

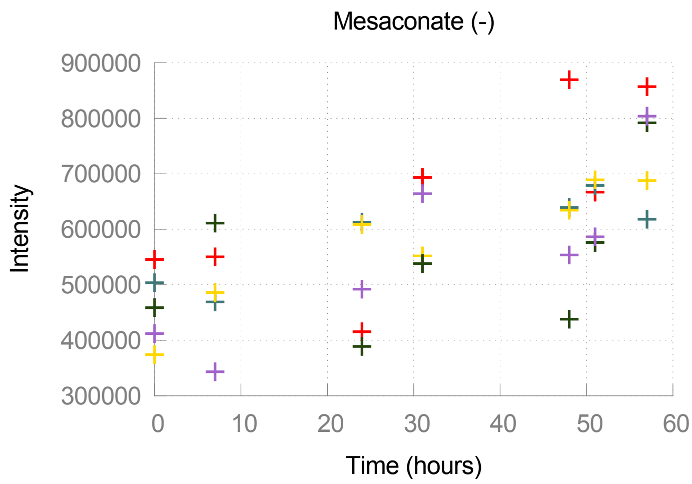
Mesaconate (-)
Intensity
900000
800000
700000
600000
500000
400000
300000
0
10
20
30
40
50
60
Time (hours)

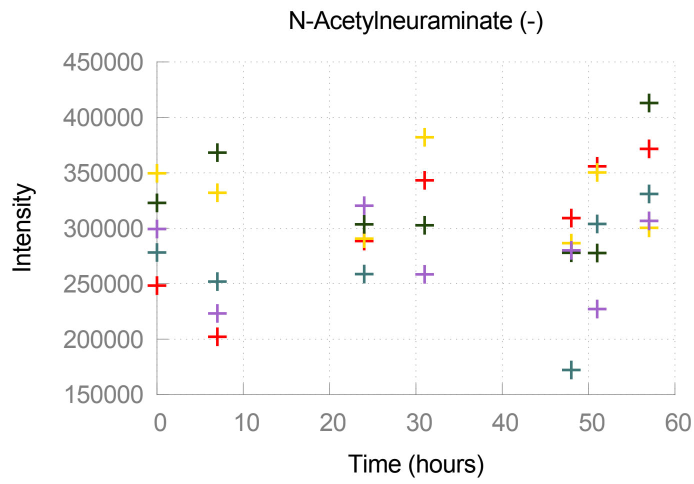
N-Acetylneuraminate (-)
Intensity
450000
400000
350000
300000
250000
200000
150000
0
10
20
30
40
50
60
Time (hours)

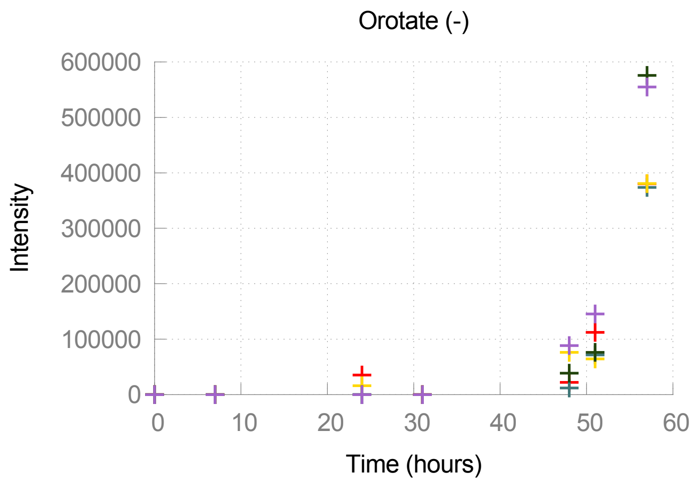
Orotate (-)
Intensity
600000
500000
400000
300000
200000
100000
0
0
10
20
30
40
50
60
Time (hours)

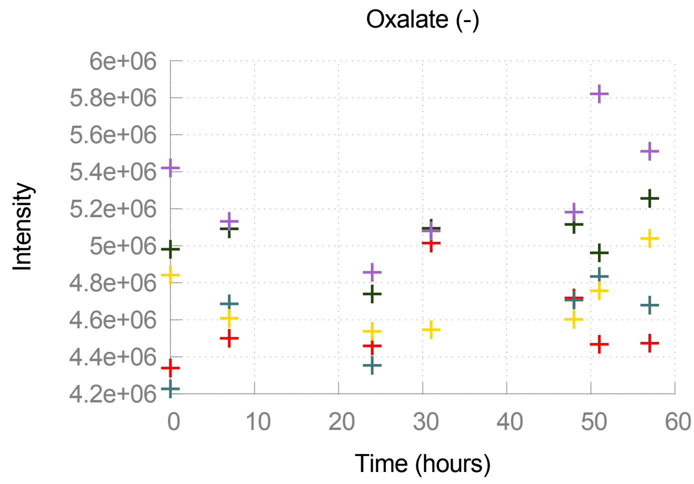
Oxalate (-)
Intensity
6e+06
5.8e+06
5.6e+06
5.4e+06
5.2e+06
5e+06
4.8e+06
4.6e+06
4.4e+06
4.2e+06
0
10
20
30
40
50
60
Time (hours)

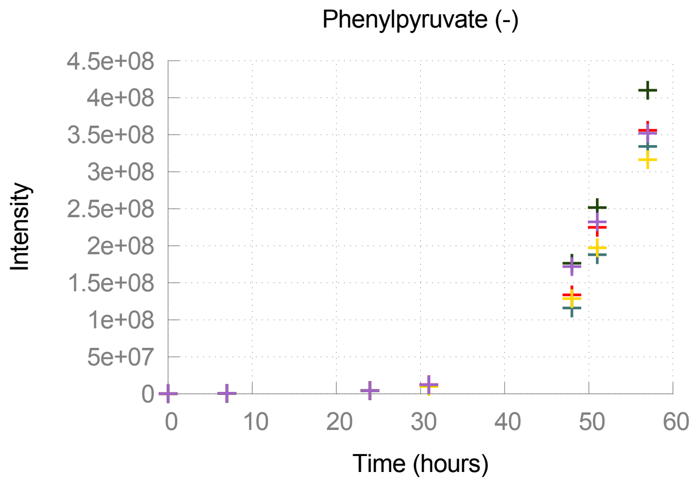
Phenylpyruvate (-)
Intensity
4.5e+08
4e+08
3.5e+08
3e+08
2.5e+08
2e+08
1.5e+08
1e+08
5e+07
0
0
10
20
30
40
50
60
Time (hours)

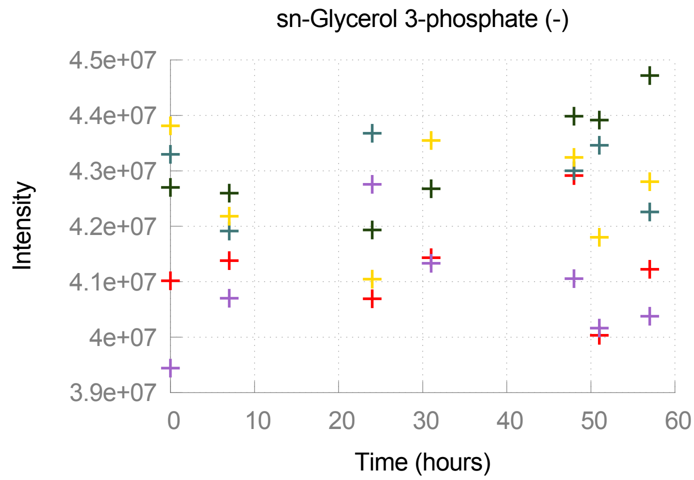
sn-Glycerol 3-phosphate (-)
Intensity
4.5e+07
4.4e+07
4.3e+07
4.2e+07
4.1e+07
4e+07
3.9e+07
0
10
20
30
40
50
60
Time (hours)

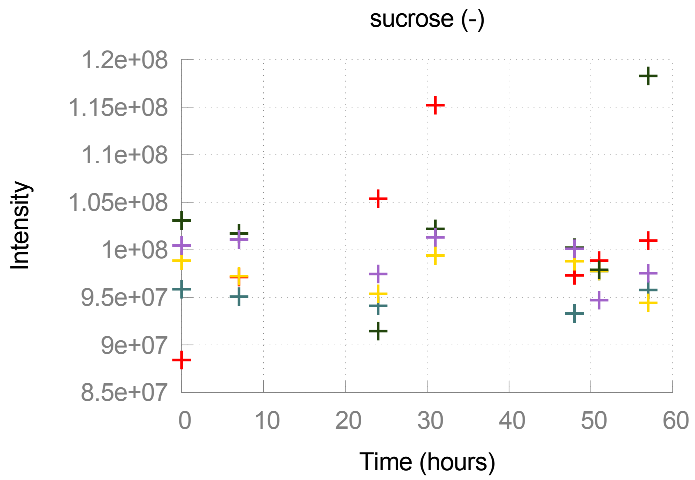
sucrose (-)
Intensity
1.2e+08
1.15e+08
1.1e+08
1.05e+08
1e+08
9.5e+07
9e+07
8.5e+07
0
10
20
30
40
50
60
Time (hours)
