# Supplementary material for: LC–MS-based absolute metabolite quantification: application to metabolic flux measurement in trypanosomes
Source: Metabolomics. 2015 Jul 9;11(6):1721–32. doi: 10.1007/s11306-015-0827-2 (PMC4605981; doi:10.1007/s11306-015-0827-2)
Supplement: Supplementary file 6 — Supplementary material 6 (PDF 123 kb). Supplementary figure S7 – PDF of growths of 5 independent cultures of T. brucei bloodstream form grown in CMM (+) and HMI-9 (×). Open and closed circle: growth of two independent cultures of T. brucei bloodstream form of Haanstra et al. (2012) [file 11306_2015_827_MOESM6_ESM.pdf]

SUPPLEMENTARY INFORMATION

LC-MS-based absolute metabolite quantification: Application to metabolic flux measurement in trypanosomes

Dong-Hyun Kim[1,2], Fiona Achcar[1], Rainer Breitling[3], Karl E. Burgess[4], Michael P. Barrett[1,4*]

[1]Wellcome Trust Centre for Molecular Parasitology, Institute of Infection, Immunity and Inflammation, College of Medical Veterinary and Life Sciences, University of Glasgow, G12 8TA, UK

[2]Current: Centre for Analytical Bioscience, School of Pharmacy, University of Nottingham, University Park, Nottingham, NG7 2RD, UK

[3]Manchester Centre of Synthetic Biology for Fine and Speciality Chemicals, Manchester Institute of Biotechnology, Faculty of Life Sciences, University of Manchester, Manchester, M1 7DN, UK.

[4]Glasgow Polyomics, Wolfson Wohl Cancer Research Centre, College of Medical Veterinary & Life Sciences, University of Glasgow, G61 1QH, UK

*Address for Correspondence:

M.P. Barrett, Wellcome Trust Centre for Molecular Parasitology, Institute of Infection, Immunity and Inflammation, College of Medical Veterinary and Life Sciences, University of Glasgow, G12 8TA, UK
E-mail: Michael.Barrett@glasgow.ac.uk
Tel: +44 (0) 141 330 6904. Fax: +44 (0) 141 330 4077

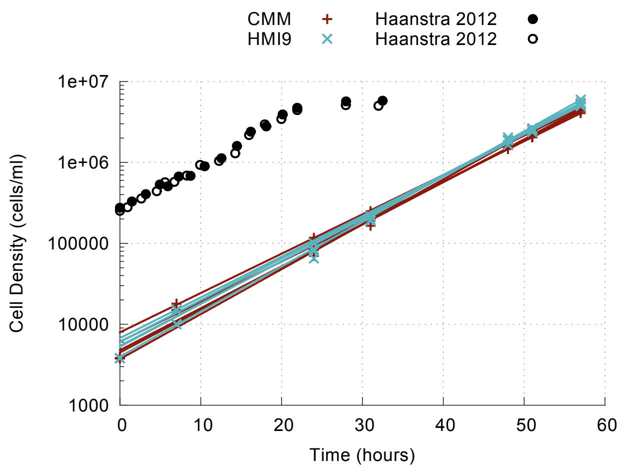

CMM
HMI9
Haanstra 2012
Haanstra 2012
1e+07
1e+06
100000
10000
1000
Cell Density (cells/ml)
0
10
20
30
40
50
60
Time (hours)
